# A versatile genetic toolkit for engineering *Wickerhamomyces ciferrii* for tetraacetyl phytosphingosine production

**DOI:** 10.3389/fbioe.2025.1586218

**Published:** 2025-04-28

**Authors:** Seong-Rae Lee, Jun Su Kang, Pyung Cheon Lee

**Affiliations:** Department of Molecular Science and Technology and Advanced College of Bio-convergence Engineering, Ajou University, Suwon, Republic of Korea

**Keywords:** genetic toolkit, non-model yeast, *Wickerhamomyces ciferrii*, episomal plasmid system, tetraacetyl phytosphingosine

## Abstract

*Wickerhamomyces ciferrii*: a non-conventional yeast with significant industrial potential for tetraacetyl phytosphingosine (TAPS), remains underutilized due to the lack of a comprehensive genetic toolbox. In this study, we developed a modular genetic system tailored for *Wickerhamomyces ciferrii* to enable strain engineering and metabolic pathway optimization. This toolkit includes episomal plasmids incorporating multiple selectable markers, replication origins, and fluorescent reporters. Systematic evaluation of four antibiotic resistance markers demonstrated that nourseothricin, geneticin, and zeocin effectively confer resistance, whereas hygromycin B did not support selection in this host. Among three tested replication origins, 2μ and CEN6/ARS4 enabled stable episomal maintenance, whereas panARS failed to replicate. Expression analysis of six fluorescent proteins under the endogenous *PGK1* promoter revealed significant variability in transcript levels, which correlated with codon adaptation index values, emphasizing the importance of codon optimization for heterologous expression. Additionally, characterization of the endogenous *TDH3, PGK1,* and *PDA1* promoters using two highly expressed fluorescent proteins confirmed that promoter strength is largely independent of the downstream coding sequence. To demonstrate the functional application of this toolkit, we overexpressed a phosphorylation-insensitive mutant of acetyl-CoA carboxylase (*ACC1* ^*S26A-S1161A*^), resulting in a 2.4-fold increase in TAPS production. Collectively, this study establishes a versatile genetic platform for *W. ciferrii*, providing a robust foundation for future synthetic biology and metabolic engineering applications.

## Introduction

The increasing demand for sustainable and scalable bioproduction systems has driven interest in microbial platforms as alternatives to traditional chemical synthesis (Patra et al., 2021; Ploessl et al., 2023). Yeasts are particularly attractive due to their eukaryotic cellular machinery, metabolic versatility, and ability to perform post-translational modifications, making them well-suited for industrial biotechnology (Nielsen, 2013). While *Saccharomyces cerevisiae* is the most extensively studied yeast, its industrial applicability is often limited by stress sensitivity, inefficient precursor accumulation, and rigid metabolic constraints (Fraczek et al., 2018; Liang et al., 2024; Singh et al., 2022; Utomo et al., 2021). Consequently, non-conventional yeasts with enhanced stress tolerance and unique biosynthetic capabilities are increasingly being explored as alternative chassis organisms (Deparis et al., 2017; Lacerda et al., 2020; Walker and Basso, 2020; Zhou et al., 2025; Baptista and Domingues, 2022; Cao et al., 2022; Duman-Özdamar and Binay, 2021; Liu et al., 2023; Ma et al., 2022; Manfrão-Netto et al., 2019; Park and Ledesma-Amaro, 2023; Qiao et al., 2017; Sun et al., 2020; Tran et al., 2019).

Among these, *W. ciferrii* (formerly *Hansenula ciferri* and later *Pichia ciferrii*) is notable for its native ability to synthesize tetraacetyl phytosphingosine (TAPS), a high-value sphingolipid precursor with applications in cosmetics and pharmaceuticals (Wickerham and Stodola, 1960; Barenholz et al., 1971). Since TAPS shares biosynthetic pathways with other sphingolipids, including sphinganine and sphingosine, *Wickerhamomyces ciferrii* presents a promising platform for engineering sphingolipid biosynthesis (Eun and Lee, 2023). Early metabolic engineering strategies have focused on the overexpression of *LCB2*, disruption of negative regulatory genes (*ORM12*, *LCB4*), and optimization of serine metabolism, leading to significant improvements in TAPS yield (Bae et al., 2003; Börgel et al., 2012; Schorsch et al., 2012). More recently, genome sequencing, adaptive laboratory evolution, and gamma-ray mutagenesis have facilitated further strain improvements, culminating in the highest reported TAPS titer of 20.2 g/L in fed-batch fermentation (Schneider et al., 2012; Choi et al., 2021; Yoo et al., 2023).

In addition to its native sphingolipid biosynthetic capacity, *W. ciferrii* possesses several desirable traits as a chassis for synthetic biology. It is among the fast-growing yeast species, with a reported specific growth rate of 0.27–0.64 h^-1^ under defined conditions (Wronska et al., 2020). Indeed, *W. ciferrii* is already used for commercial-scale production of ceramide-related compounds for skincare (Yoo et al., 2023; Schorsch et al., 2012; Pérez-Rivero and López-Gómez, 2023). These features, along with its demonstrated tractability in fermentation processes, support its potential as a safe and efficient microbial host for biomanufacturing.

Despite these advancements, the lack of a well-developed genetic toolkit remains a major barrier to *W. ciferrii*’s broader adoption in synthetic biology (Schorsch et al., 2009). Currently, available tools are limited to a small set of antibiotic selection markers and replication origins, restricting the flexibility of plasmid-based genetic manipulation. Expanding the repertoire of replication elements, fluorescent reporters, and tunable promoters would greatly improve strain engineering efforts, allowing for more precise pathway optimization and metabolic control.

**FIGURE 1:**
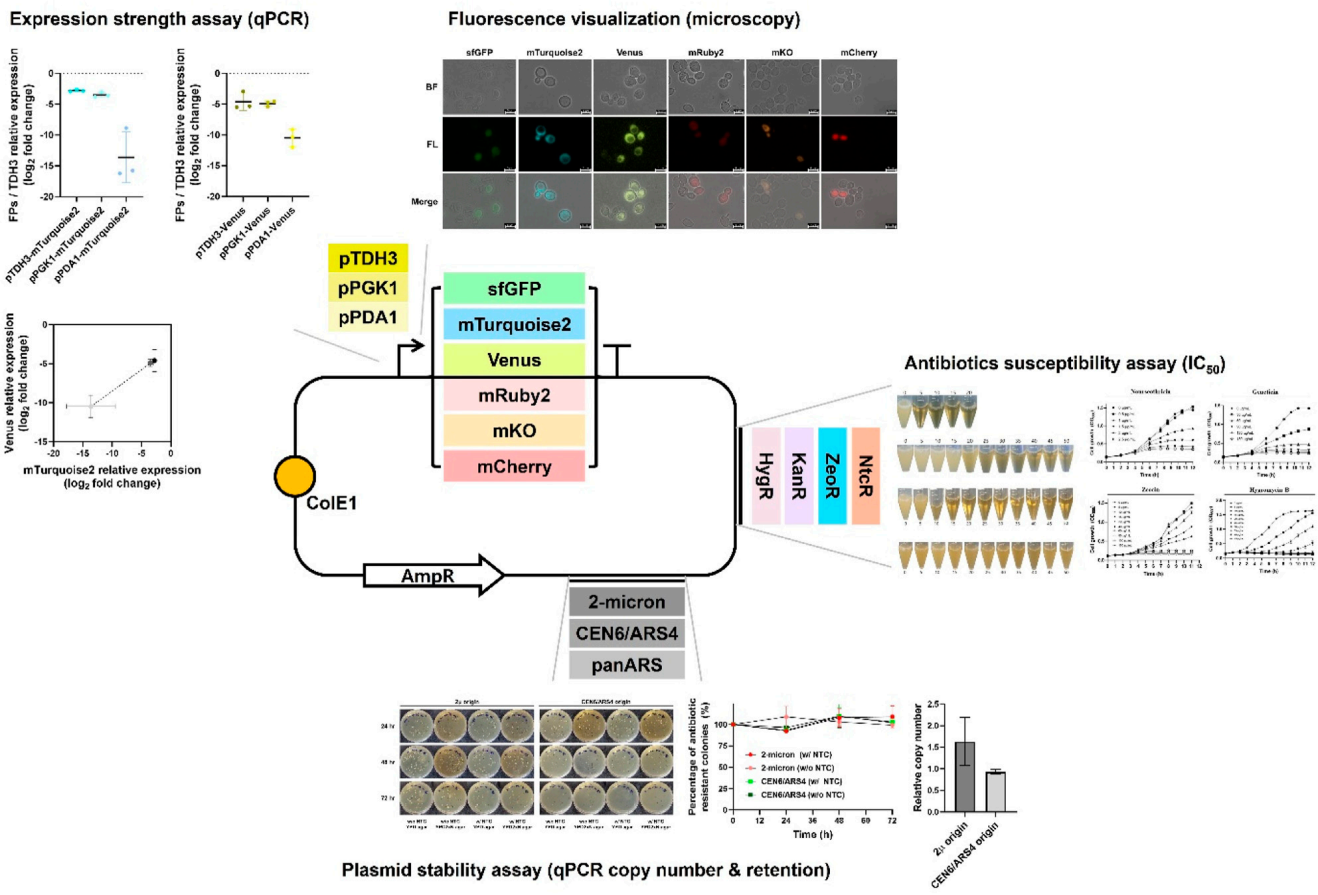
Overview of the genetic toolkit construction and functional characterization in *Wickerhamomyces ciferrii*. This schematic illustrates the modular plasmid system developed in this study, highlighting the key genetic elements and their experimental validation. The base plasmid contains a bacterial backbone (ColE1 origin and ampicillin resistance marker) for propagation in *Escherichia coli*. Functional modules evaluated in *Wickerhamomyces ciferrii* include: (i) three replication origins (2μ, CEN6/ARS4, panARS), compared in terms of plasmid maintenance and copy number; (ii) four antibiotic resistance markers (NtcR, ZeoR, KanR, HygR), tested for selection efficiency on both solid and liquid media; (iii) six fluorescent protein genes (sfGFP, mTurquoise2, Venus, mRuby2, mKO, mCherry), evaluated by fluorescence microscopy and transcript analysis; (iv) three endogenous promoters (pTDH3, pPGK1, pPDA1), assessed for transcriptional strength via quantitative real-time PCR (qPCR). Representative experimental results for each functional module are summarized adjacent to the schematic.

To address these limitations, this study aims to systematically characterize and expand the genetic toolkit for *W. ciferrii* by evaluating four antibiotic selection markers, three replication origins, and six fluorescent proteins. Additionally, three endogenous promoters—pTDH3, pPGK1, and pPDA1—were assessed for their strength and expression variability (Figure 1). Finally, to validate the functionality of this toolkit, we overexpressed a phosphorylation-insensitive mutant of acetyl-CoA carboxylase (*ACC1* ^S26A−S1161A^), demonstrating its impact on TAPS biosynthesis. Together, these findings provide a versatile molecular platform for future strain engineering, metabolic optimization, and synthetic biology applications in *W. ciferrii*.

## Material and methods

### Strains, media, and growth conditions

*Wickerhamomyces ciferrii* (F-60-10A NRRL 1031) wild-type strain was used for the overall study. Single colonies were obtained by streaking the cell stock on YPD agar (10 g/L yeast extract, 20 g/L peptone, 20 g/L glucose, and 1.5% agar) and incubating at 25°C for 2 days. For 96-well plate cultivation in an antibiotic IC_50_ test, single colonies were inoculated into 200 μL of YPD medium (10 g/L yeast extract, 20 g/L peptone, and 20 g/L glucose) and aerobically cultured at 25°C with shaking at 800 rpm. For short-term cultivation prior to genomic DNA extraction, fluorescence analysis, plasmid copy number determination, transcriptional expression analysis, and main culture inoculation, single colonies were inoculated into test tubes containing 2 mL of YPD medium (with or without appropriate antibiotics) and incubated at 25°C with shaking at 250 rpm for 12–24 h. Cells were subsequently harvested and processed for each respective assay. For long-term cultivation, test tube-scale pre-cultured cells were inoculated into 250 mL Erlenmeyer flasks containing 50 mL of YMglSC medium at an initial OD_600_ of 0.1 and cultured at 25°C, 250 rpm until the carbon source was depleted. Appropriate antibiotics were added to the culture medium at concentrations to be determined in this study. Cell growth was monitored at a wavelength of 600 nm (OD_600_) with a SPECTRAmax PLUS384 spectrophotometer (Molecular Devices, United States). For plasmid cloning and propagation, *Escherichia coli* NEB 10-beta (New England Biolabs Korea, Republic of Korea) was aerobically grown overnight in Luria-Bertani (LB) medium (10 g/L tryptone, 5 g/L yeast extract, and 5 g/L NaCl) at 37°C with shaking at 250 rpm. Solid medium was prepared by adding 1.5% agar to the recipe. For selection, 100 μg/mL ampicillin or 50 μg/mL chloramphenicol was added to the solid agar plates and culture tubes containing 4 mL of LB medium.

### Genomic DNA extraction

Genomic DNA of *W. ciferrii* was extracted following the protocol of Dymond et al., with slight modifications (Dymond, 2013). Briefly, a single colony generated from YPD agar was inoculated into 2 mL of YPD medium in a test tube and incubated overnight at 25°C with shaking at 250 rpm. One milliliter of the culture was transferred to a 1.7 mL microcentrifuge tube and centrifuged at 14,000 rpm for 1 min at room temperature. After removing the supernatant, the cell pellet was resuspended in 200 μL of yeast lysis buffer [10 mM Tris-HCl (pH 8.0), 1 mM EDTA (pH 8.0), 100 mM NaCl, 2% Triton X-100, 1% sodium dodecyl sulfate (SDS)]. Next, 150 μL of glass beads and 200 μL of phenol/chloroform/isoamyl alcohol (25:24:1, v/v/v) were added to the suspension, followed by vigorous vortexing for 10 min. Subsequently, 400 μL of TE buffer [10 mM Tris-HCl (pH 8.0), 1 mM EDTA (pH 8.0)] was added, and the mixture was vortexed for one additional minute. After centrifugation at 4°C and 13,000 rpm for 10 min, the upper aqueous layer was transferred to a new 1.7 mL microcentrifuge tube. An equal volume of isopropyl alcohol was added and gently inverted to mix. After centrifugation at 4°C and 13,000 rpm for 10 min, a pellet was observed. The pellet was washed with 1 mL of chilled 70% ethanol. After discarding the supernatant by centrifugation at 4°C and 13,000 rpm for 5 min, the pellet was air-dried at room temperature for approximately 5 min. Finally, the dried pellet was resuspended in 100 μL of TE buffer and used for subsequent polymerase chain reaction (PCR).

### Plasmid construction and sequencing

Plasmids and strains used in this study are listed in Table 1, and primers used for PCR are provided in Table 2. Plasmid were constructed using Golden Gate assembly or Gibson assembly. DNA fragments used for assembly were amplified by PCR, with detailed information on the fragments and their corresponding primers listed in Table 3. Oligonucleotides for PCR were obtained from Macrogen (Seoul, Republic of Korea), and PCR was performed using Phusion High-Fidelity DNA polymerase (New England Biolabs Korea, Republic of Korea) following the manufacturer’s manual. The DNA fragments used for assembly were gel-purified with a gel extraction kit (GeneAll, Republic of Korea).

**TABLE 1:** Plasmids and strains used in this study.

Plasmid/strain	Description	Source
Plasmid		
pYTK002	MoClo-YTK plasmid kit (ConLS linker)	Addgene
pYTK032	MoClo-YTK plasmid kit (mTurquoise2 gene)	Addgene
pYTK033	MoClo-YTK plasmid kit (Venus gene)	Addgene
pYTK034	MoClo-YTK plasmid kit (mRuby2 gene)	Addgene
pYTK048	MoClo-YTK plasmid kit (spacer)	Addgene
pYTK067	MoClo-YTK plasmid kit (ConR1 linker)	Addgene
pYTK077	MoClo-YTK plasmid kit (geneticin resistance marker)	Addgene
pYTK078	MoClo-YTK plasmid kit (nourseothricin resistance marker)	Addgene
pYTK079	MoClo-YTK plasmid kit (hygromycin resistance marker)	Addgene
pYTK080	MoClo-YTK plasmid kit (zeocin resistance marker)	Addgene
pYTK081	MoClo-YTK plasmid kit (CEN6/ARS4 origin sequence)	Addgene
pYTK082	MoClo-YTK plasmid kit (2μ origin sequence)	Addgene
pYTK083	MoClo-YTK plasmid kit (bacterial vector backbone part: ampicillin resistance marker and *E. coli* ColE1 origin	Addgene
p2μ_WcNAT	Amp^R^, ColE1 ori, 2μ ori, nourseothricin resistance marker, Gibson assembly of F1, F2, F3, F4, and F5	This study
pCEN_WcNAT	Amp^R^, ColE1 ori, CEN6/ARS4 ori, nourseothricin resistance marker, Gibson assembly of F1, F2, F3, F4, and F6	This study
ppan_WcNAT	Amp^R^, ColE1 ori, panARS ori, nourseothricin resistance marker, Gibson assembly of F1, F2, F3, F4, and F7	This study
p2μ_NTC	Amp^R^, ColE1 ori, 2μ ori, nourseothricin resistance marker, Golden Gate assembly of pYTK002, pYTK048, pYTK067, pYTK078, pYTK082, and pYTK083	This study
pCEN_NTC	Amp^R^, ColE1 ori, CEN6/ARS4 ori, nourseothricin resistance marker, Golden Gate assembly of pYTK002, pYTK048, pYTK067, pYTK078, pYTK081, and pYTK083	This study
p2μ_G418	Amp^R^, ColE1 ori, 2μ ori, geneticin resistance marker, Golden Gate assembly of pYTK002, pYTK048, pYTK067, pYTK077, pYTK082, and pYTK083	This study
p2μ_Zeo	Amp^R^, ColE1 ori, 2μ ori, zeocin resistance marker, Golden Gate assembly of pYTK002, pYTK048, pYTK067, pYTK080, pYTK082, and pYTK083	This study
p2μ_HYG	Amp^R^, ColE1 ori, 2μ ori, hygromycin resistance marker, Golden Gate assembly of pYTK002, pYTK048, pYTK067, pYTK079, pYTK082, and pYTK083	This study
p2μ_pPGK1-sfGFP_NTC	Amp^R^, ColE1 ori, 2μ ori, nourseothricin resistance marker, pPGK1-sfGFP-tENO1, Golden Gate assembly of pYTK002, F8, F9, F12, pYTK067, pYTK078, pYTK082, and pYTK083	This study
p2μ_pPGK1-mTurquoise2_NTC	Amp^R^, ColE1 ori, 2μ ori, nourseothricin resistance marker, pPGK1-mTurquoise2-tENO1, Golden Gate assembly of pYTK002, F8, pYTK032, F12, pYTK067, pYTK078, pYTK082, and pYTK083	This study
p2μ_pPGK1-Venus_NTC	Amp^R^, ColE1 ori, 2μ ori, nourseothricin resistance marker, pPGK1-Venus-tENO1, Golden Gate assembly of pYTK002, F8, pYTK033, F12, pYTK067, pYTK078, pYTK082, and pYTK083	This study
p2μ_pPGK1-mRuby2_NTC	Amp^R^, ColE1 ori, 2μ ori, nourseothricin resistance marker, pPGK1-mRuby2-tENO1, Golden Gate assembly of pYTK002, F8, pYTK034, F12, pYTK067, pYTK078, pYTK082, and pYTK083	This study
p2μ_pPGK1-mKO_NTC	Amp^R^, ColE1 ori, 2μ ori, nourseothricin resistance marker, pPGK1-mKO-tENO1, Golden Gate assembly of pYTK002, F8, F10, F12, pYTK067, pYTK078, pYTK082, and pYTK083	This study
p2μ_pPGK1-mCherry_NTC	Amp^R^, ColE1 ori, 2μ ori, nourseothricin resistance marker, pPGK1-mCherry-tENO1, Golden Gate assembly of pYTK002, F8, F11, F12, pYTK067, pYTK078, pYTK082, and pYTK083	This study
pCEN_pPGK1-sfGFP_NTC	Amp^R^, ColE1 ori, CEN6/ARS4 ori, nourseothricin resistance marker, pPGK1-sfGFP-tENO1, Golden Gate assembly of pYTK002, F8, F9, F12, pYTK067, pYTK078, pYTK081, and pYTK083	This study
pCEN_pPGK1-mTurquoise2_NTC	Amp^R^, ColE1 ori, CEN6/ARS4 ori, nourseothricin resistance marker, pPGK1-mTurquoise2-tENO1, Golden Gate assembly of pYTK002, F8, pYTK032, F12, pYTK067, pYTK078, pYTK081, and pYTK083	This study
pCEN_pPGK1-Venus_NTC	Amp^R^, ColE1 ori, CEN6/ARS4 ori, nourseothricin resistance marker, pPGK1-Venus-tENO1, Golden Gate assembly of pYTK002, F8, pYTK033, F12, pYTK067, pYTK078, pYTK081, and pYTK083	This study
pCEN_pPGK1-mRuby2_NTC	Amp^R^, ColE1 ori, CEN6/ARS4 ori, nourseothricin resistance marker, pPGK1-mRuby2-tENO1, Golden Gate assembly of pYTK002, F8, pYTK034, F12, pYTK067, pYTK078, pYTK081, and pYTK083	This study
pCEN_pPGK1-mKO_NTC	Amp^R^, ColE1 ori, CEN6/ARS4 ori, nourseothricin resistance marker, pPGK1-mKO-tENO1, Golden Gate assembly of pYTK002, F8, F10, F12, pYTK067, pYTK078, pYTK081, and pYTK083	This study
pCEN_pPGK1-mCherry_NTC	Amp^R^, ColE1 ori, CEN6/ARS4 ori, nourseothricin resistance marker, pPGK1-mCherry-tENO1, Golden Gate assembly of pYTK002, F8, F11, F12, pYTK067, pYTK078, pYTK081, and pYTK083	This study
p2μ_pPDA1-mTurquoise2_NTC	Amp^R^, ColE1 ori, 2μ ori, nourseothricin resistance marker, pPDA1-mTurquoise2-tENO1, Gibson assembly of F14 and F18	This study
p2μ_pTDH3-mTurquoise2_NTC	Amp^R^, ColE1 ori, 2μ ori, nourseothricin resistance marker, pTDH3-mTurquoise2-tENO1, Gibson assembly of F14 and F16	This study
p2μ_pPDA1-Venus_NTC	Amp^R^, ColE1 ori, 2μ ori, nourseothricin resistance marker, pPDA1-Venus-tENO1, Gibson assembly of F13 and F17	This study
p2μ_pTDH3-Venus_NTC	Amp^R^, ColE1 ori, 2μ ori, nourseothricin resistance marker, pPDA1-Venus-tENO1, Gibson assembly of F13 and F15	This study
p2μ_pTDH3-ACC1mut_NTC	Amp^R^, ColE1 ori, 2μ ori, nourseothricin resistance marker, pTDH3-ACC1mut-tENO1, Gibson assembly of F19, F21, F22, and F23	This study
p2μ_pPDA1-ACC1mut_NTC	Amp^R^, ColE1 ori, 2μ ori, nourseothricin resistance marker, pPDA1-ACC1mut-tENO1, Gibson assembly of F20, F21, F22, and F24	This study

Strain	Description	References
*Escherichia coli* 10-beta	*Δ(ara-leu) 7,697 araD139 fhuA ΔlacX74 galK16 galE15 e14- Φ80dlacZΔM15 recA1 relA1 endA1 nupG rpsL (StrR) rph spoT1 Δ(mrr-hsdRMS-mcrBC)*	NEB
*Wickerhamomyces ciferrii* F-60-10 A NRRL1031	Wild-type strain	Wickerham and Stodola (1960)
*W.c_p2μ_WcNAT*	Wild-type *W. ciferrii* harboring p2μ_WcNAT	This study
*W.c_pCEN_WcNAT*	Wild-type *W. ciferrii* harboring pCEN_WcNAT	This study
*W.c_ppan_WcNAT*	Wild-type *W. ciferrii* harboring ppan_WcNAT	This study
*W.c_2μ-sfGFP-mod*	Wild-type *W. ciferrii* harboring p2μ_pPGK1-sfGFP_NTC	This study
*W.c_2μ-mTurq2-mod*	Wild-type *W. ciferrii* harboring p2μ_pPGK1-mTurquoise2_NTC	This study
*W.c_2μ-Venus-mod*	Wild-type *W. ciferrii* harboring p2μ_pPGK1-Venus_NTC	This study
*W.c_2μ-mRuby2-mod*	Wild-type *W. ciferrii* harboring p2μ_pPGK1-mRuby2_NTC	This study
*W.c_2μ-mKO-mod*	Wild-type *W. ciferrii* harboring p2μ_pPGK1-mKO_NTC	This study
*W.c_2μ-mCherry-mod*	Wild-type *W. ciferrii* harboring p2μ_pPGK1-mCherry_NTC	This study
*W.c_CEN-sfGFP-mod*	Wild-type *W. ciferrii* harboring pCEN_pPGK1-sfGFP_NTC	This study
*W.c_CEN-mTurq2-mod*	Wild-type *W. ciferrii* harboring pCEN_pPGK1-mTurquoise2_NTC	This study
*W.c_CEN-Venus-mod*	Wild-type *W. ciferrii* harboring pCEN_pPGK1-Venus_NTC	This study
*W.c_CEN-mRuby2-mod*	Wild-type *W. ciferrii* harboring pCEN_pPGK1-mRuby2_NTC	This study
*W.c_CEN-mKO-mod*	Wild-type *W. ciferrii* harboring pCEN_pPGK1-mKO_NTC	This study
*W.c_CEN-mCherry-mod*	Wild-type *W. ciferrii* harboring pCEN_pPGK1-mCherry_NTC	This study
*W.c_2μ-mTurq2-str*	Wild-type *W. ciferrii* harboring p2μ_pTDH3-mTurquoise2_NTC	This study
*W.c_2μ-Venus-str*	Wild-type *W. ciferrii* harboring p2μ_pTDH3-Venus_NTC	This study
*W.c_2μ-mTurq2-weak*	Wild-type *W. ciferrii* harboring p2μ_pPDA1-mTurquoise2_NTC	This study
*W.c_2μ-Venus-weak*	Wild-type *W. ciferrii* harboring p2μ_pPDA1-Venus_NTC	This study
*W.c_2μ-ev*	Wild-type *W. ciferrii* harboring p2μ_NTC	This study
*W.c_2μ-ACC1m-str*	Wild-type *W. ciferrii* harboring p2μ_pTDH3-ACC1mut_NTC	This study
*W.c_2μ-ACC1m-weak*	Wild-type *W. ciferrii* harboring p2μ_pPDA1-ACC1mut_NTC	This study

The fragments (F1, F2, etc.) used in Gibson assembly and Golden Gate assembly were amplified by PCR., The corresponding primers used for amplification are listed in Table 3.

**TABLE 2:** Primers used in this study.

Oligo name	Sequence (5′- 3′)
Gibson_pPDA1_F	TGCTCTAAATTTGCCCGG
Gibson_pPDA1_R	TGA​TAA​TAA​AGT​TGA​TTT​TGA​AGT​TTG
Gibson_nat_F	CAA​AAT​CAA​CTT​TAT​TAT​CAA​TGG​GTA​CTA​CTT​TAG​ATG​AC
Gibson_nat_R	GGA​AAG​CAC​CGA​AGC​TAA​ATG​ATC​CTT​ATG​GAC​ATG​GC
Gibson_tENO1_F	ATT​TAG​CTT​CGG​TGC​TTT​CC
Gibson_tENO1_R	TTA​TAA​CGG​TTG​GGC​AAT​G
Gibson_backbone_F	ACA​TTG​CCC​AAC​CGT​TAT​AAC​GGC​CGC​GAT​TAT​CAA​AAA​G
Gibson_backbone_R	CAC​GGT​TAT​CCA​CAG​AAT​CAG
Gibson_2mi_F	CTG​ATT​CTG​TGG​ATA​ACC​GTG​AAC​GAA​GCA​TCT​GTG​CTT​C
Gibson_2mi_R	AAC​CGG​GCA​AAT​TTA​GAG​CAG​AAG​TTC​CTA​TTC​TCT​AGC​TAG
Gibson_CENARS_F	CTG​ATT​CTG​TGG​ATA​ACC​GTG​GGT​CTC​AGA​GTA​TCA​CGT​G
Gibson_CENARS_R	AAC​CGG​GCA​AAT​TTA​GAG​CAG​ACG​GAT​CGC​TTG​CCT​G
Gibson_panARS_F	CTG​ATT​CTG​TGG​ATA​ACC​GTG​CAA​CAT​CTT​TGG​ATA​ATA​TCA​G
Gibson_panARS_R	AAC​CGG​GCA​AAT​TTA​GAG​CAT​AGT​GCT​GAT​TAT​GAT​TTG​ACG
Golden gate_pPGK1_F	GGT​CTC​AAA​CGG​TCA​CAA​GTT​GGT​GCA​ATC
Golden gate_pPGK1_R	GGT​CTC​ACA​TAC​TTG​ATG​TGT​TAG​TAA​TGG​GT
Golden gate_sfGFP_F	GGT​CTC​ATA​TGA​TGA​GCA​AAG​GAG​AAG​AAC
Golden gate_sfGFP_R	GGT​CTC​AGG​ATT​CAT​TTG​TAG​AGC​TCA​TCC​ATG
Golden gate_mKO_F	GGT​CTC​ATA​TGG​TTT​CAG​TTA​TTA​AGC​CG
Golden gate_mKO_R	GGT​CTC​AGG​ATT​CAA​GAG​TGT​GCG​ACA​GC
Golden gate_mCherry_F	GGT​CTC​ATA​TGG​TAA​GTA​AAG​GCG​AGG
Golden gate_mCherry_R	GGT​CTC​AGG​ATT​CAC​TTA​TAC​AAC​TCA​TCC
Golden gate_tENO1_F	GGT​CTC​AAT​CCA​TTT​AGC​TTC​GGT​GCT​TTC
Golden gate_tENO1_R	GGT​CTC​ACA​GCT​TAT​AAC​GGT​TGG​GCA​ATG​T
Gibson_Venus_F	ATG​TCT​AAA​GGT​GAA​GAA​TTA​TTC
Gibson_mTurq2_F	ATG​GTT​TCT​AAA​GGT​GAA​GAA​T
Gibson_bbEX_R	CGT​TCG​TCT​GCA​ATT​ATC​GGC
Gibson_pTDH3_F	CCG​ATA​ATT​GCA​GAC​GAA​CGG​GAC​CGT​TAA​TTA​CCA​ACA​ATC
Gibson_pTDH3-Venus_R	TAA​TTC​TTC​ACC​TTT​AGA​CAT​ATG​TTA​ATT​ATT​TGT​TTG​TTT​GTT​TG
Gibson_pTDH3-mTurq2_R	ATT​CTT​CAC​CTT​TAG​AAA​CCA​TAT​GTT​AAT​TAT​TTG​TTT​GTT​TGT​TTG
Gibson_pPDA1_F	CCG​ATA​ATT​GCA​GAC​GAA​CGT​GCT​CTA​AAT​TTG​CCC​GG
Gibson_pPDA1-Venus_R	ATA​ATT​CTT​CAC​CTT​TAG​ACA​TAT​GAT​AAT​AAA​GTT​GAT​TTT​GAA​GTT​TG
Gibson_pPDA1-mTurq2_R	ATT​CTT​CAC​CTT​TAG​AAA​CCA​TAT​GAT​AAT​AAA​GTT​GAT​TTT​GAA​GTT​TG
ACC1_S26A_F	GAT​TTA​CAT​AAC​GCA​ATA​CCT​TC
ACC1_S26A_R	GAA​GGT​ATT​GCG​TTA​TGT​AAA​TC
ACC1_S1161A_F	GGT​AGA​GCT​GTT​GCA​GTT​TCT​G
ACC1_S1161A_R	CAG​AAA​CTG​CAA​CAG​CTC​TAC​C
Gibson_ACC1_pTDH3_F	AAA​CAA​ACA​AAT​AAT​TAA​CAA​TGA​GTG​AAG​AAA​ATT​TGA​GTG​AAG​TTG
Gibson_ACC1_pPDA1_F	CAA​AAT​CAA​CTT​TAT​TAT​CAA​TGA​GTG​AAG​AAA​ATT​TGA​GTG​AAG​TTG
Gibson_ACC1_R	ATG​AGC​CGT​GAT​GAC​CCC​GTC​ATA​ATT​CCA​ATC​TAC​AAT​GCA​ATC
Gibson_bb_ACC1_F	ACGGGGTCATCACGGC
Gibson_bb_pTDH3_R	TGT​TAA​TTA​TTT​GTT​TGT​TTG​TTT​G
qPCR_nat_F	CCT​GAG​CAT​AGA​GGT​CAC​GG
qPCR_nat_R	TTG​CTC​ACC​GTC​ACT​AGC​AG
qPCR_sfGFP_F	CGC​CAC​AAC​GTT​GAA​GAT​GG
qPCR_sfGFP_R	ATG​CCA​TGT​GTA​ATC​CCA​GCA
qPCR_mTurq2_F	AGA​CAC​AAC​ATT​GAA​GAT​GGT​GG
qPCR_mTurq2_R	TCC​ATA​CCC​AAG​GTA​ATA​CCA​G
qPCR_Venus_F	AGA​CAC​AAC​ATT​GAA​GAT​GGT​GG
qPCR_Venus_R	ATG​GGT​AAT​ACC​AGC​AGC​AGT
qPCR_mRuby2_F	AGA​GGC​TAC​ACA​CAC​ATG​GC
qPCR_mRuby2_R	CTA​CGG​CAT​GCT​CTC​TCT​G
qPCR_mKO_F	CGG​CTT​CTG​ATG​GCG​TTT​TG
qPCR_mKO_R	TGC​GAC​AGC​ATC​TTC​AAC​CAA
qPCR_mCherry_F	TAA​TTT​CCC​CTC​CGA​CGG​C
qPCR_mCherry_R	TGC​ACC​GGG​CAA​TTG​TAC​TG
qPCR_TDH3_F	GCT​GCT​AAA​GCT​GTT​GGT​AAA​G
qPCR_TDH3_R	AAC​CAA​CAA​CAC​CTT​TTA​ATG​GAC
qPCR_ACC1_F	GAT​GTT​GAT​GCA​GTT​TGG​GCT
qPCR_ACC1_R	ACA​CCG​GTA​CCA​GAC​CAA​G

**TABLE 3:** PCR fragment used for assembly in this study.

PCR primer	PCR template	PCR product	No.
Gibson_pPDA1_F	*W. ciferrii* genomic DNA	Frag_pPDA1_Gib	F1
Gibson_pPDA1_R			
Gibson_nat_F	Lab plasmid	Frag_nat_Gib	F2
Gibson_nat_R			
Gibson_tENO1_F	*W. ciferrii* genomic DNA	Frag_tENO1_Gib	F3
Gibson_tENO1_R			
Gibson_backbone_F	pYTK083 (MoClo-YTK plasmid kit)	Frag_bb_Gib	F4
Gibson_backbone_R			
Gibson_2mi_F	pYTK082 (MoClo-YTK plasmid kit)	Frag_2mi_Gib	F5
Gibson_2mi_R			
Gibson_CENARS_F	pYTK081 (MoClo-YTK plasmid kit)	Frag_CENARS_Gib	F6
Gibson_CENARS_R			
Gibson_panARS_F	*Kluyveromyces lactis* genomic DNA	Frag_panARS_Gib	F7
Gibson_panARS_R			
Golden gate_pPGK1_F	*W. ciferrii* genomic DNA	Frag_pPGK1_GG	F8
Golden gate_pPGK1_R			
Golden gate_sfGFP_F	Lab plasmid	Frag_sfGFP_GG	F9
Golden gate_sfGFP_R			
Golden gate_mKO_F	Lab plasmid	Frag_mKO_GG	F10
Golden gate_mKO_R			
Golden gate_mCherry_F	Lab plasmid	Frag_mCherry_GG	F11
Golden gate_mCherry_R			
Golden gate_tENO1_F	*W. ciferrii* genomic DNA	Frag_tENO1_GG	F12
Golden gate_tENO1_R			
Gibson_Venus_F	p2μ_pPGK1-Venus_NTC	Frag_Venus-bb_Gib	F13
Gibson_bbEX_R			
Gibson_mTurq2_F	p2μ_pPGK1-mTurquoise2_NTC	Frag_mTurq2-bb_Gib	F14
Gibson_bbEX_R			
Gibson_pTDH3_F	*W. ciferrii* genomic DNA	Frag_pTDH3-Venus_Gib	F15
Gibson_pTDH3-Venus_R			
Gibson_pTDH3_F	*W. ciferrii* genomic DNA	Frag_pTDH3-mTurq2_Gib	F16
Gibson_pTDH3-mTurq2_R			
Gibson_pPDA1_F	*W. ciferrii* genomic DNA	Frag_pPDA1-Venus_Gib	F17
Gibson_pPDA1-Venus_R			
Gibson_pPDA1_F	*W. ciferrii* genomic DNA	Frag_pPDA1-mTurq2_Gib	F18
Gibson_pPDA1-mTurq2_R			
Gibson_ACC1_TDH3p_F	*W. ciferrii* genomic DNA	Frag_pTDH3-ACC1.1_Gib	F19
ACC1_S26A_R			
Gibson_ACC1_PDA1p_F	*W. ciferrii* genomic DNA	Frag_pPDA1-ACC1.1_Gib	F20
ACC1_S26A_R			
ACC1_S26A_F	*W. ciferrii* genomic DNA	Frag_ACC1.2_Gib	F21
ACC1_S1161A_R			
ACC1_S1161A_F	*W. ciferrii* genomic DNA	Frag_ACC1.3_Gib	F22
Gibson_ACC1_R			
Gibson_bb_ACC1_F	p2μ_pTDH3-Venus_NTC	Frag_bb-pTDH3_Gib	F23
Gibson_bb_pTDH3_R			
Gibson_bb_ACC1_F	p2μ_pPDA1-Venus_NTC	Frag_bb-pPDA1_Gib	F24
Gibson_pPDA1_R			

The MoClo-YTK plasmid kit was used to obtain four antibiotic resistance marker sequences (nourseothricin, geneticin, zeocin, and hygromycin B), each expressed under the *Ashbya gossypii TEF* promoter and *TEF* terminator, three fluorescent protein coding genes (mTurquoise2, Venus, and mRuby2), two origins of replication commonly used in *Saccharomyces cerevisiae* (2μ and CEN6/ARS4), and a bacterial vector backbone containing the ampicillin resistance marker and ColE1 origin of replication for selection in *E. coli* (Lee et al., 2015). The MoClo-YTK plasmid kit was a gift from John Dueber (Addgene kit # 1000000061).

To identify a replication origin sequence capable of supporting stable plasmid replication in *W. ciferrii*, plasmids p2μ_WcNAT, pCEN_WcNAT, and ppan_WcNAT were constructed by assembling one of three replication origin sequences—2μ and CEN6/ARS4 from *S. cerevisiae*, and panARS from *Kluyveromyces lactis*—with a *nat* marker cassette and a bacterial vector backbone. The 2μ and CEN6/ARS4 sequences were obtained from pYTK082 and pYTK081 of MoClo-YTK plasmid kit, and panARS was obtained from genomic DNA of *K. lactis*. This *nat* marker cassette consisted of the *W. ciferrii* endogenous pyruvate dehydrogenase subunit E1 alpha (*PDA1*) promoter, a codon-optimized *nat* gene, and the *W. ciferrii* endogenous enolase (*ENO1*) terminator. The assembly was performed using Gibson Assembly Master Mix (New England Biolabs Korea, Republic of Korea). The *PDA1* promoter and the *ENO1* terminator sequence were identified using the reference genome sequence available in the NCBI database. Specifically, the upstream 675 bp region of the start codon was extracted from the annotated reference sequence (Gene ID: BN7_3959a, RefSeq: NW_011887617.1) for the *PDA1* promoter, and the downstream 332 bp region of the stop codon was extracted from the reference sequence (Gene ID: BN7_149, RefSeq: NW_011887739.1) for the *ENO1* terminator.

Next, for screening of selectable markers applicable to *W. ciferrii*, four additional plasmids—p2μ_NTC, p2μ_G418, p2μ_ZEO, and p2μ_HYG—were constructed. Four marker cassettes expressed under the *A. gossypii TEF* promoter and *TEF* terminator were amplified by PCR from the MoClo-YTK plasmid kit (pYTK078, pYTK077, pYTK080, and pYTK079, respectively). Each amplified cassette was then cloned via Gibson assembly, together with the 2μ origin of replication sequence amplified from pYTK082 and the bacterial vector backbone from pYTK083.

For the construction of fluorescent protein expression vectors used in functional reporter protein screening, Golden Gate cloning was performed following the protocol by Michael E. Lee et al. to generate twelve plasmids carrying either 2μ or CEN6/ARS4 replication origin: p2μ_pPGK1-sfGFP_NTC, p2μ_pPGK1-mTurquoise2_NTC, p2μ_pPGK1-Venus_NTC, p2μ_pPGK1-mRuby2_NTC, p2μ_pPGK1-mKO_NTC, p2μ_pPGK1-mCherry_NTC, pCEN_pPGK1-sfGFP_NTC, pCEN_pPGK1-mTurquoise2_NTC, pCEN_pPGK1-Venus_NTC, pCEN_pPGK1-mRuby2_NTC, pCEN_pPGK1-mKO_NTC, and pCEN_pPGK1-mCherry_NTC (Lee et al., 2015). The plasmid assembly required eight parts: (1) linker, (2) promoter, (3) gene, (4) terminator, (5) linker, (6) yeast selectable marker, (7) yeast origin of replication, and (8) bacterial vector backbone. To incorporate the *W. ciferrii* endogenous phosphoglycerate 1 (*PGK1*) promoter and *ENO1* terminator into positions (2) and (4), respectively, PCR amplification was carried out using *W. ciferrii* genomic DNA as the template with primers containing BsaⅠ restriction sites along with appropriate flanking sequences to ensure compatibility with adjacent parts. The *PGK1* gene sequence was identified from the NCBI database (Gene ID: BN7_3735, RefSeq: NW_011887629.1), and the 497 bp upstream region of the start codon was used as the promoter. To further expand the fluorescent protein library beyond mTurquoise2, Venus, and mRuby2 from the MoClo-YTK plasmid kit, additional fluorescent proteins, including superfolder GFP (sfGFP), mKO, and mCherry, were amplified using the same primer design strategy and incorporated into part (3). For the assembly, PCR-amplified parts: (2) *PGK1* promoter, (3) sfGFP, mKO, and mCherry genes, and (4) *ENO1* terminator were used, while the remaining parts were directly obtained from the MoClo-YTK plasmid kit. The specific part numbers used in the assembly are listed in Table 3. Each part was mixed equimolarly (20 fmol) in a single tube together with 20 U of BsaⅠ, 400 U of T4 DNA ligase, 2 μL of T4 DNA ligase buffer (all from New England Biolabs Korea, Republic of Korea), and deionized water to a final volume of 20 μL. The reaction was carried out under the following conditions [37°C for 5 min, 16°C for 5 min] × 30 cycles, followed by 60°C for 5 min, and an infinite hold at 4°C.

To compare fluorescence intensity based on promoter strength, three representative endogenous promoters commonly used in yeast research were selected: the glyceraldehyde 3-phosphate dehydrogenase promoter (pTDH3), the phosphoglycerate kinase 1 promoter (pPGK1), and the pyruvate dehydrogenase subunit E1 alpha promoter (pPDA1). Using these promoters, four additional plasmids—p2μ_pPDA1-mTurquoise2_NTC, p2μ_pTDH3-mTurquoise2_NTC, p2μ_pPDA1-Venus_NTC, and p2μ_pTDH3-Venus_NTC—were constructed in addition to the two previously generated plasmids carrying the *PGK1* promoter, p2μ_pPGK1-mTurquoise2_NTC and p2μ_pPGK1-Venus_NTC. The promoter sequences were identified based on the reference genome of *W. ciferrii* available in the NCBI database: *TDH3* gene (Gene ID: BN7_5327, RefSeq: NW_011887532.1), *PGK1* gene (Gene ID: BN7_3735, RefSeq: NW_011887629.1), *PDA1* gene (Gene ID: BN7_3959a, RefSeq: NW_011887617.1). The upstream 418 bp (pTDH3), 497 bp (pPGK1), and 675 bp (pPDA1) regions of the start codon were extracted and used as the endogenous promoter parts. These three promoters, amplified from *W. ciferrii* genomic DNA by PCR, were individually cloned with both mTurquoise2 and Venus, which were the most highly expressed fluorescent proteins in *W. ciferrii*, using Gibson assembly together with the endogenous *ENO1* terminator, the nourseothricin resistance marker, the 2μ origin of replication, and the bacterial vector backbone.

To construct an *ACC1* mutant expression vector for practical application in enhancing tetraacetyl phytosphingosine (TAPS) production, the *W. ciferrii* ACC1 sequence was identified through a BLASTp search, using the amino acid sequence of *Rattus norvegicus* (rat) ACC1, retrieved from UniProtKB/Swiss-Prot (Accession: P11497.1) as the query. To enhance enzyme activity, site-directed mutagenesis was performed on the *W. ciferrii ACC1* gene sequence to introduce the S26A and S1161A mutations, as previously reported. For vector construction, the *ACC1* ^S26A−S1161A^ gene, including the coding sequence and the terminator region (from the start codon to 225 bp downstream of the stop codon), was amplified by PCR. The resulting fragment was then cloned using Gibson assembly, incorporating two different promoters (pTDH3 for strong expression and pPDA1 for weak expression), the ACC1^S26A−S1161A^ coding sequence and terminator, the nourseothricin resistance marker, the 2μ origin of replication, and the bacterial vector backbone parts in the designated order, resulting in the p2μ_pTDH3-ACC1(mut)_NTC and p2μ_pPDA1-ACC1(mut)_NTC plasmids.

The sequences of all constructed vectors were confirmed by Sanger sequencing (Macrogen, Republic of Korea).

### Transformation of *Wickerhamomyces ciferrii* and *Escherichia coli*

The Golden Gate and Gibson assembly mixtures, prepared as described above, were transformed into *E*. *coli* 10-beta competent cells (New England Biolabs Korea, Republic of Korea) via electroporation following the manufacturer’s protocol. The cloned plasmids were then introduced into *W. ciferrii* using a slightly modified version of a previously published protocol (Bae et al., 2003).

A single *W. ciferrii* colony from YPD agar was inoculated into 2 mL of YPD medium and cultured until reaching an OD_600_ of 0.8–1.2. The cells were harvested by centrifugation and resuspended in 0.1 culture volume of 50 mM phosphate buffer (pH 7.5) supplemented with 25 mM dithiothreitol (DTT). After shaking incubation at 37°C for 15 min, the cells were washed twice with one culture volume of STM solution (10 mM Tris-HCl [pH 7.5], 270 mM sucrose, 1 mM MgCl_2_) and finally resuspended in 0.01 culture volume of STM solution. The prepared competent cells were aliquoted into 50-μL portions and stored at −80°C until use.

For transformation, 50 μL of frozen *W. ciferrii* competent cells were mixed with ∼1 μg of plasmid DNA and kept on ice until fully thawed. The mixture was transferred to a 0.2-cm electroporation cuvette (Bio-Rad, United States), and electroporation was performed using a GenePulser Xcell™ (Bio-Rad, United States) at 500 V, 50 μF, and 700 Ω. Following electroporation, cells were immediately resuspended in 500 μL of STM solution and transferred to a test tube containing 2 mL of YPD medium. After incubation at 25°C and 250 rpm for 6–12 h, the cells were plated onto YPD agar containing the appropriate antibiotic. Colonies were observed after 3 days of incubation at 25°C.

### Screening of functional origin of replication

To assess whether plasmids could be maintained and confer resistance in *W. ciferrii*, three plasmids, p2μ_WcNAT, pCEN_WcNAT, and ppan_WcNAT, each carrying a replication origin—2μ and CEN6/ARS4 from *S. cerevisiae* and panARS from *K. lactis*—along with the nourseothricin resistance marker and the bacterial vector backbone, were transformed into *W. ciferrii* competent cells, which were then plated onto YPD2xN agar (YPD medium containing 100 μg/mL nourseothricin). Colony formation was assessed after incubation at 25°C for 3 days.

To evaluate plasmid stability over culture time, transformants obtained from YPD2xN agar were inoculated into 2 mL of YPDN liquid medium (YPD containing 50 μg/mL nourseothricin) and cultured at 25°C, 250 rpm for 12 h. The overnight culture was then transferred into 50 mL of YMglSCN medium (YMglSC medium containing 50 μg/mL nourseothricin) in a 250 mL baffled flask at an initial OD_600_ = 0.1 and incubated for 72 h. Every 24 h, equal numbers of cells were plated onto YPD agar (without antibiotics) and YPD2xN agar (with antibiotics), and the percentage of antibiotic-resistant colonies was calculated.

### Antibiotic and selectable marker screening

To screen for antibiotics and selectable markers applicable to the wild-type diploid *W. ciferrii* strain, four commonly used antibiotics in yeast research were tested at various concentrations to assess cell viability. Cells were pre-cultured in 2 mL of YPD medium in test tubes and incubated at 25°C, 250 rpm for 12 h. After incubation, cultures were inoculated at 1% (v/v) into 200 µL of YPD medium containing different antibiotic concentrations in a 96-well plate. Since *W. ciferrii* exhibited varying susceptibility to each antibiotic, multiple trials were conducted to determine the appropriate concentration ranges: nourseothricin (0–2.5 μg/mL), geneticin (0–45 μg/mL), zeocin (0–45 μg/mL), and hygromycin B (0–45 μg/mL). Cell growth was monitored by measuring OD_600_ at 1-h intervals, and statistical analysis was performed using biological triplicates. The specific growth rate of *W. ciferrii* was calculated using the formula “ln (OD_2_/OD_1_)/(t_2_-t_1_)”. The obtained values were normalized to the untreated antibiotic set, and concentrations of antibiotics inhibiting 50% of the growth were determined.

In order to identify selectable markers that confer effective resistance, four plasmids—p2μ_NTC, p2μ_G418, p2μ_ZEO, and p2μ_HYG—carrying each antibiotic resistance marker sequence (nourseothricin, geneticin, zeocin, and hygromycin B) from the MoClo-YTK plasmid kit, along with the previously constructed p2μ_WcNAT plasmid were transformed into *W. ciferrii* competent cells and spread onto YPD agar containing the respective antibiotics to assess colony formation. Antibiotic concentrations were systematically optimized by incrementally increasing from previously reported values for various yeast species—nourseothricin (50 μg/mL), geneticin (200 μg/mL), zeocin (100 μg/mL), and hygromycin B (100 μg/mL) (Bai et al., 2021; Chen et al., 2016; Schorsch et al., 2009; Zhao et al., 2020)—to minimize false-positive colonies while maintaining effective selection.

### Relative plasmid copy number determination

To determine the relative plasmid copy number in *W. ciferrii*, a quantitative real-time PCR (qPCR) was performed using the *TDH3* gene as a reference and the nourseothricin resistance marker (*nat*) gene as the target. Transformants were generated by transforming *W. ciferrii* competent cells with p2u_NTC or pCEN_NTC plasmids, each carrying the nourseothricin resistance marker and either the 2μ origin or CEN6/ARS4 origin. Single colonies were isolated from YPD2xN agar plates, and five single colonies from each plate were inoculated into test tubes containing 2 mL of YPDN medium. Cultures were incubated at 25°C and 250 rpm overnight. Following incubation, cells were harvested by centrifugation, and genomic DNA was extracted. qPCR was performed on genomic DNA extracted from a total of 10 transformant samples (five from each plasmid type) using the SensiFAST™ SYBR No-ROX One-Step Kit (Bioline, United States). For each sample, both the *TDH3* reference gene and the *nat* target gene were amplified, and Ct values were obtained using a Rotor-Gene system (Qiagen, Germany). Relative plasmid copy number was determined using the ΔCt method, where ΔCt = Ct (*nat*) - Ct (*TDH3*). Since *W. ciferrii* is diploid, the *TDH3* gene copy number was set to 2, and relative plasmid copy number was calculated as 2^−ΔCt^ × 2. The sequences of primers used—qPCR_nat_F, qPCR_nat_R, qPCR_TDH3_F, and qPCR_TDH3_R—are listed in Table 2.

### Fluorescence analysis

For fluorescence observation in *W. ciferrii*, plasmids expressing six different fluorescent proteins—sfGFP, mTurquoise2, Venus, mRuby2, mKO, and mCherry—were introduced into *W. ciferrii* competent cells via transformation. Single colonies selected on YPD2xN agar plates were inoculated into test tubes containing 2 mL of YPDN medium, followed by incubation at 25°C and 250 rpm overnight. Cultures typically reached OD_600_ ≈ 4 after overnight incubation, which corresponds to the mid-logarithmic phase of *W. ciferrii* growth. After cultivation, cells were harvested by centrifugation and washed twice with 1× phosphate-buffered saline (PBS) to remove residual medium. Fluorescence microscopy was performed using a LEICA DM2500 microscope (LEICA, Germany) equipped with a LEICA DFC450 C digital camera. To visualize the fluorescence signals, two different filter sets were used depending on the excitation and emission spectra of each fluorescent protein. The FITC filter set (LEICA L5 filter) was used for sfGFP (488/510 nm), mTurquoise2 (434/474 nm), and Venus (515/528 nm), while the TRITC filter set (LEICA N2.1 filter) was used for mRuby2 (559/600 nm), mKO (548/559 nm), and mCherry (587/610 nm).

### Transcriptional gene expression analysis

To assess the transcriptional expression levels of heterologous genes in *W. ciferrii* transformants, cells were cultivated at 25°C and 250 rpm until reaching the logarithmic growth phase. At this stage, a portion of the culture corresponding to an OD_600_ of approximately 10 was harvested by centrifugation. The supernatant was completely removed, and the cell pellet was resuspended in 1 mL of RNAlater stabilization solution (Thermo Fisher, United States) to preserve RNA integrity. Total RNA was extracted from the harvested cells using the easy-BLUE™ Total RNA Extraction Kit (iNtRON Biotechnology, Republic of Korea) following the manufacturer’s instructions. Complementary DNA (cDNA) was synthesized using the ReverTraAce™ qPCR RT Kit (Toyobo, Japan). Quantitative reverse transcription PCR (qRT-PCR) was subsequently performed using the SensiFAST™ No-ROX One-Step Kit (Bioline, United States) on a Rotor-Gene (Qiagen, Germany). For each cDNA sample, two separate qRT-PCR reactions were conducted: one for the gene of interest and one for the reference gene *TDH3*. Relative transcriptional expression levels were analyzed using both the ΔCt and ΔΔCt methods. In the ΔCt method, ΔCt was calculated as Ct (target) - Ct (*TDH3*), with *TDH3* serving as the reference gene. For comparative expression analysis across strains, the ΔΔCt method was applied, where the ΔCt values of the target gene in overexpression strains were normalized to its ΔCt value in the designated negative control strain. Primer sequences used for amplification are listed in Table 2, specifically those designated as ‘qPCR_gene_F’ and ‘qPCR_gene_R’.

### Extraction and analysis of TAPS

Recombinant *W. ciferrii* strains were cultivated in 50 mL of YMglSCN medium in 250 mL baffled flasks. Cultures were incubated at 25°C and 250 rpm until glycerol depletion in triplicate. For TAPS quantification, 1 mL of each culture was mixed with 4 mL of methanol, followed by vigorous vortexing for 30 min. After extraction, the mixture was centrifuged, and the resulting supernatant was filtered through a 0.2 μm PTFE syringe membrane filter. The filtrate was then analyzed using high-performance liquid chromatography (HPLC) on an Agilent 1,260 system (Agilent Technology, United States) equipped with a photodiode array detector and a ZORBAX Eclipse XDB-C18 column (150 mm × 4.6 mm, 5 μm, Agilent Technology) at 20°C. Gradient elution was performed at a flow rate of 1 mL/min with mobile phase A (100% acetonitrile) and mobile phase B (100% deionized distilled water, DDW) under the following conditions: 45%–80% A (0–18 min), 80%–90% A (18–20 min), 90%–100% A (20–23 min), 100% A (23–25 min), 100%–45% A (25–28 min), and 45% A (28–30 min). TAPS was quantified at 200 nm based on peak area values, using a standard curve established in this study.

### Codon adaptation index (CAI) calculation and correlation analysis

To evaluate the potential impact of codon usage on gene expression in *W. ciferrii*, the codon adaptation index (CAI) was calculated using the online tool CAIcal (http://genomes.urv.es/CAIcal/) with codon usage tables generated via the Countcodon program (https://www.kazusa.or.jp/codon/) (Nakamura et al., 2000; Puigbò et al., 2008). The reference gene set for CAI calculation consisted of ten genes involved in the sphingoid base biosynthetic pathway of *W. ciferrii*: *ATF2* (NCBI Gene ID: BN7_5921, RefSeq: NW_011887500.1), *LCB1* (NCBI Gene ID: BN7_169, RefSeq: NW_011887738.1), *LCB2* (NCBI Gene ID: BN7_5771, RefSeq: NW_011887508.1), *LCB3* (NCBI Gene ID: BN7_3163, RefSeq: NW_011887655.1), *LCB4* (NCBI Gene ID: BN7_4658, RefSeq: NW_011887561.1), *ORM1* (NCBI Gene ID: BN7_3097, RefSeq: NW_011887657.1), *SLI1* (NCBI Gene ID: BN7_6322, RefSeq: NW_011887476.1), *SYR2* (NCBI Gene ID: BN7_5855, RefSeq: NW_011887504.1), *TSC3* (NCBI Gene ID: BN7_3864, RefSeq: NW_011887620.1), and *TSC10* (NCBI Gene ID: BN7_3057, RefSeq: NW_011887660.1).

CAI values were computed for six fluorescent proteins (sfGFP, mTurquoise2, Venus, mRuby2, mKO, and mCherry) and four antibiotic resistance markers (nourseothricin, zeocin, geneticin, and hygromycin B) obtained from the MoClo-YTK plasmid kit. To examine the relationship between codon adaptation and transcript levels, linear regression analysis was performed using CAI values as the independent variable (x-axis) and the relative transcriptional expression levels of the fluorescent proteins (log_2_ fold change of FP/TDH3) as the dependent variable (y-axis). Each data point represents biological triplicates of transcript levels for each fluorescent protein. The mKO dataset was excluded from this analysis due to its undetectable transcript levels. The correlation between the relative transcriptional expression levels of mTurquoise2 and Venus under different promoter strengths was also analyzed, with mTurquoise2 plotted on the x-axis and Venus on the y-axis. The correlation coefficient (*R* ^*2*^) and statistical significance (*p*-value) were determined.

### Statistical analysis

All data are presented as mean ± standard deviation (SD) from 3 or 5 biological replicates. Linear regression analysis and unpaired t-test with Welch’s correction were performed using GraphPad Prism (version 8.0.2). Statistical significance was defined as *p* < 0.05 (ns: *p* ≥ 0.05, *p < 0.05, **p < 0.01, ***p < 0.001).

## Results

### Screening and verification of replication origins for stable episomal maintenance in *Wickerhamomyces ciferrii*

The availability of autonomously replicating plasmids in *W. ciferrii* remains largely unexplored. To date, the only reported sequence enabling episomal maintenance in *W. ciferrii* is the *S. cerevisiae* CEN/ARS origin (Yoo et al., 2023). However, no additional replication origins have been systematically characterized, and most genetic studies have relied on integrative vectors for gene expression and genome modifications (Bae et al., 2003; Börgel et al., 2012; Schorsch et al., 2009; 2012). To address this limitation, we aimed to establish a versatile plasmid system incorporating alternative replication origins for efficient gene expression in *W. ciferrii*.

**FIGURE 2:**
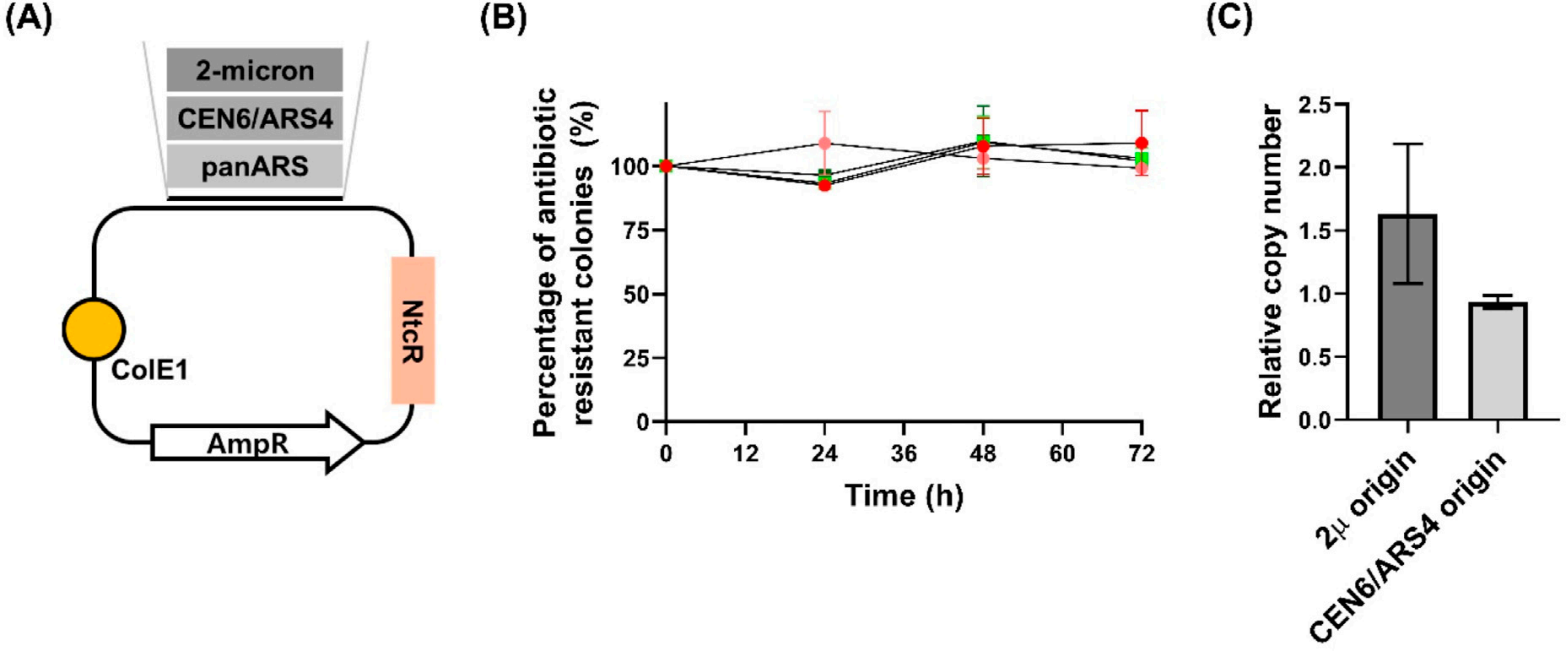
Stability and copy number analysis of plasmids with different origins of replication in *Wickerhamomyces ciferrii* **(A)** Schematic representation of the three constructed plasmids. Each plasmid harbors a distinct candidate origin of replication (2μ, CEN6/ARS4, or panARS) and includes a nourseothricin resistance (NtcR) marker functional in *Wickerhamomyces ciferrii* **(B)** Long-term segregational stability assay. Transformants were cultivated in both selective and non-selective media, and at designated time points (0, 24, 48, and 72 h), equal numbers of cells were plated onto both antibiotic-containing and antibiotic-free agar plates. The percentage of antibiotic-resistant colonies was calculated to assess plasmid retention. Data are presented as mean ± standard deviation (SD) from three biological replicates (n = 3) **(C)** Quantification of relative plasmid copy number. The relative copy numbers of plasmids carrying the 2μ and CEN6/ARS4 origins were determined via quantitative PCR. Values were normalized to the genomic *TDH3* gene, which was used as a diploid reference (copy number = 2). Data represent the mean ± SD from three biological replicates (n = 3).

We selected three replication origins: the 2μ origin and CEN6/ARS4 sequence, both widely used in *S. cerevisiae*, and the panARS sequence from *K. lactis*, suggested as a broad-host-range origin (Camattari et al., 2016; Liachko and Dunham, 2014) (Figure 2A). Plasmids carrying these origins—p2μ_WcNAT (2μ origin), pCEN_WcNAT (CEN6/ARS4 origin), and ppan_WcNAT (panARS origin)—were transformed into *W. ciferrii* and plated onto selective YPD2xN agar. After 3 days at 25°C, colonies formed for *W. c_p2μ_WcNAT* and *W. c_pCEN_WcNAT*, whereas no growth was observed for *W. c_ppan_WcNAT*, indicating that panARS does not support episomal replication in *W. ciferrii*.

To confirm plasmid stability, single colonies of *W. c_p2μ_WcNAT* and *W. c_pCEN_WcNAT* were grown in YPDN liquid medium (YPD supplemented with 50 μg/mL nourseothricin) for 12 h, showing normal growth. Long-term plasmid stability was further assessed by cultivating both strains for 72 h in YMglSC medium (without selection) and YMglSCN medium (with 50 μg/mL nourseothricin). At 24-h intervals, equal numbers of cells were plated onto selective and non-selective YPD agar. After 3 days at 25°C, CFU counts showed no significant differences across time points, indicating stable plasmid retention even after 72 h without selection (Figure 2B). Additionally, quantitative PCR (qPCR) analysis revealed that plasmids with the 2μ origin exhibited a higher copy number than those with the CEN6/ARS4 origin (Figure 2C), reinforcing the difference in replication properties.

Collectively, these findings establish that 2μ and CEN6/ARS4 support stable episomal replication in *W. ciferrii*, enabling long-term plasmid maintenance without selection. This work expands the genetic toolkit for *W. ciferrii*, providing foundational resources for strain engineering and synthetic biology applications.

### Evaluation of antibiotic susceptibility and selectable markers in *Wickerhamomyces ciferrii*

The range of antibiotic selection markers available for *W. ciferrii* remains limited, with previous studies primarily utilizing nourseothricin, geneticin, cycloheximide (via ribosomal protein L41 mutation), syringomycin E (for *SYR2* mutant screening), and 5-fluoroorotic acid (for *URA3*-based counter-selection) (Bae et al., 2003; Börgel et al., 2012; Schorsch et al., 2009; Yoo et al., 2023). However, no studies have systematically evaluated the half-maximal inhibitory concentrations (IC_50_) for these antibiotics in *W. ciferrii*. To address this gap, we assessed the susceptibility of *W. ciferrii* to four widely used antibiotics—nourseothricin, geneticin, zeocin, and hygromycin B—by culturing wild-type cells in YPD medium with increasing antibiotic concentrations and monitoring growth inhibition. The IC_50_ values were determined as follows: nourseothricin, 1.48 ± 0.12 μg/mL; geneticin, 16.52 ± 2.07 μg/mL; zeocin, 14.96 ± 1.43 μg/mL; and hygromycin B, 9.72 ± 0.65 μg/mL, indicating differential susceptibility. To evaluate whether *W. ciferrii* could acquire resistance through heterologous expression of selection markers, four plasmids—p2μ_NTC, p2μ_G418, p2μ_ZEO, and p2μ_HYG—encoding resistance genes for nourseothricin, geneticin, zeocin, and hygromycin B, respectively, were transformed into *W. ciferrii*. Transformants were plated on YPD agar supplemented with standard antibiotic concentrations (50 μg/mL nourseothricin, 200 μg/mL geneticin, 100 μg/mL zeocin, and 100 μg/mL hygromycin B). Despite strong growth inhibition in liquid culture, high levels of false positives and lawn formation were observed on three solid media (supplemented with nourseothricin, geneticin, and zeocin), suggesting that standard antibiotic concentrations were insufficient for effective selection. To improve selection efficiency, antibiotic concentrations were optimized, establishing effective selection thresholds of 100 μg/mL (solid) and 50 μg/mL (liquid) for nourseothricin, 300 μg/mL (solid) and 50 μg/mL (liquid) for geneticin, and 200 μg/mL (solid) and 100 μg/mL (liquid) for zeocin, except for hygromycin B. Under these conditions, resistant colonies were consistently recovered for nourseothricin, geneticin, and zeocin, whereas no viable hygromycin B-resistant colonies were obtained. This observed differences in selection efficiency between solid and liquid media, with lower antibiotic concentrations required for effective selection in liquid culture may be attributed to differences in antibiotic diffusion rates, microbial cell density effects, or metabolic adaptations in response to solid-phase growth. Similar phenomena have been reported previously, where structured environments such as agar surfaces reduced antibiotic efficacy due to metabolic dormancy or decreased penetration (Stokes et al., 2019). In addition, bacterial cell debris has been shown to bind and sequester antibiotics, further reducing their bioavailability in solid media (Yuan et al., 2022), thus requiring higher concentrations for effective selection.

### Determination of copy number and structural integrity of episomal plasmids in *Wickerhamomyces ciferrii*

While the 2 μ and CEN6/ARS4 origins supported stable plasmid replication in *W. ciferrii*, we quantified their retention and relative copy numbers. qPCR analysis revealed that plasmids carrying the 2 μ origin maintained a higher copy number (1.6 ± 0.59 copies per cell) than those with the CEN6/ARS4 origin (0.9 ± 0.05 copies per cell) **(** Figure 2C). These findings confirm that while both origins enable episomal maintenance, the 2 μ origin provides a greater plasmid retention advantage in *W. ciferrii*.

To verify sequence integrity, episomal plasmids were extracted from *W. ciferrii* and subsequently transformed into *E. coli* for propagation and high-quality plasmid preparation suitable for Sanger sequencing. This approach enabled confirmation that the plasmids maintained during episomal replication in *W. ciferrii* did not undergo structural rearrangements or point mutations. Both p2μ_NTC and pCEN_NTC were confirmed to be intact, reinforcing the genetic stability of episomal vectors in *W. ciferrii* and their reliability for engineering applications.

### Characterization of functional reporter fluorescent proteins in *Wickerhamomyces ciferrii*

**FIGURE 3:**
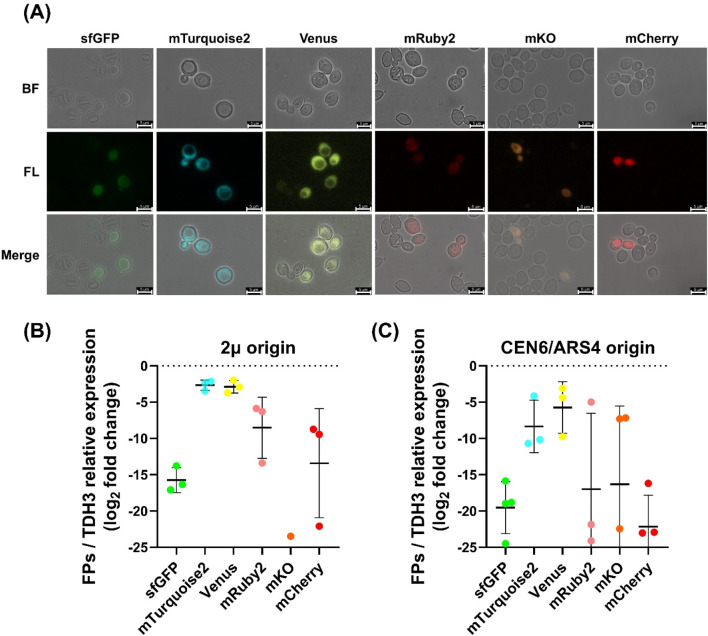
Fluorescence imaging and transcriptional analysis of fluorescent protein expression in *Wickerhamomyces ciferrii* **(A)** Fluorescence microscopy images of *Wickerhamomyces ciferrii* transformants expressing different fluorescent proteins from 2μ-based vectors. Cells harboring plasmids encoding sfGFP, mTurquoise2, Venus, mRuby2, mKO, or mCherry were imaged under bright-field (BF) and fluorescence (FL) microscopy, with merged images provided for comparison. Representative single-cell fluorescence images are shown to visualize protein-level expression from 2μ-based vectors. Fluorescence images of strains carrying CEN6/ARS4-based vectors are provided in Supplementary Data S2. Scale bars, 5 μm **(B)** Relative transcriptional expression of fluorescent protein genes in 2μ-based plasmids. Quantitative PCR (qPCR) analysis was conducted to determine mRNA levels, normalized to *TDH3* gene and presented as log_2_ fold changes. Data represent the mean ± standard deviation (SD) from three biological replicates (n = 3). Only detectable clones were analyzed for mKO **(C)** Relative transcriptional expression of fluorescent protein genes in CEN6/ARS4-based plasmids. qPCR analysis was performed as in **(B)** but for CEN6/ARS4-based vectors. Data represent the mean ± SD from three biological replicates (n = 3).

Fluorescent reporter proteins (FPs) are essential tools for monitoring gene expression, protein localization, and cellular dynamics in genetic engineering. To expand the FP toolkit for *W. ciferrii*, we evaluated six distinct FPs—sfGFP, mTurquoise2, Venus, mRuby2, mKO, and mCherry—for their expression efficiency and functionality (Figure 3A). The endogenous *PGK1* promoter (pPGK1) was selected for FP expression based on its stronger transcriptional activity compared to *PDA1* promoter (pPDA1), while avoiding overexpression artifacts (Peng et al., 2015). Twelve expression plasmids, carrying either the 2μ or CEN6/ARS4 replication origin, were generated via Golden Gate assembly and transformed into *W. ciferrii*, resulting in 12 FP-expressing strains: *W. c_2μ-sfGFP-mod, W. c_2μ-mTurq2-mod, W. c_2μ-Venus-mod, W. c_2μ-mRuby2-mod, W. c_2μ-mKO-mod, W. c_2μ-mCherry-mod, W. c_CEN-sfGFP-mod, W. c_CEN-mTurq2-mod, W. c_CEN-Venus-mod, W. c_CEN-mRuby2-mod, W. c_CEN-mKO-mod, and W. c_CEN-mCherry-mod*.

Cells for fluorescence microscopy were harvested after overnight cultivation in YPDN medium containing 50 μg/mL nourseothricin at 25°C and 250 rpm, reaching OD_600_ ≈ 4, indicative of mid-log phase of *W. ciferrii*. The fluorescence microscopy images in Figure 3A were obtained from strains carrying 2μ-based vectors and are shown as representative examples of protein-level expression. Fluorescence images of strains harboring CEN6/ARS4-based vectors are provided in Supplementary Data S2.

Following plasmid construction and transformation, we compared the performance of vectors carrying different origins of replication. Overall, 2μ-based vectors conferred more stable FP expression than CEN6/ARS4-based vectors, as evidenced by lower colony-to-colony variability in transcriptional expression levels (Figures 3B,C). Among the tested FPs, mTurquoise2 and Venus displayed the highest fluorescence intensity and expression stability, whereas sfGFP showed lower but uniform fluorescence. In contrast, mRuby2 and mCherry exhibited substantial variability, with weak fluorescence in most cells, and mKO displayed almost no detectable fluorescence. qPCR analysis corroborated these observations (Figures 3B,C). Transformants carrying 2μ-based vectors showed lower expression variability, while mTurquoise2 and Venus had the highest transcript levels, approximately 2^12^-fold and 2^11^-fold higher than sfGFP, respectively. mTurquoise2 was expressed ∼1.25-fold higher than Venus, with both showing similar trends. In contrast, mKO exhibited negligible transcript levels, consistent with its undetectable fluorescence.

These findings establish mTurquoise2 and Venus as optimal FPs for gene expression studies in *W. ciferrii*, providing valuable tools for metabolic engineering and synthetic biology applications.

### Reporter protein expression using three endogenous promoters in *Wickerhamomyces ciferrii*

**FIGURE 4:**
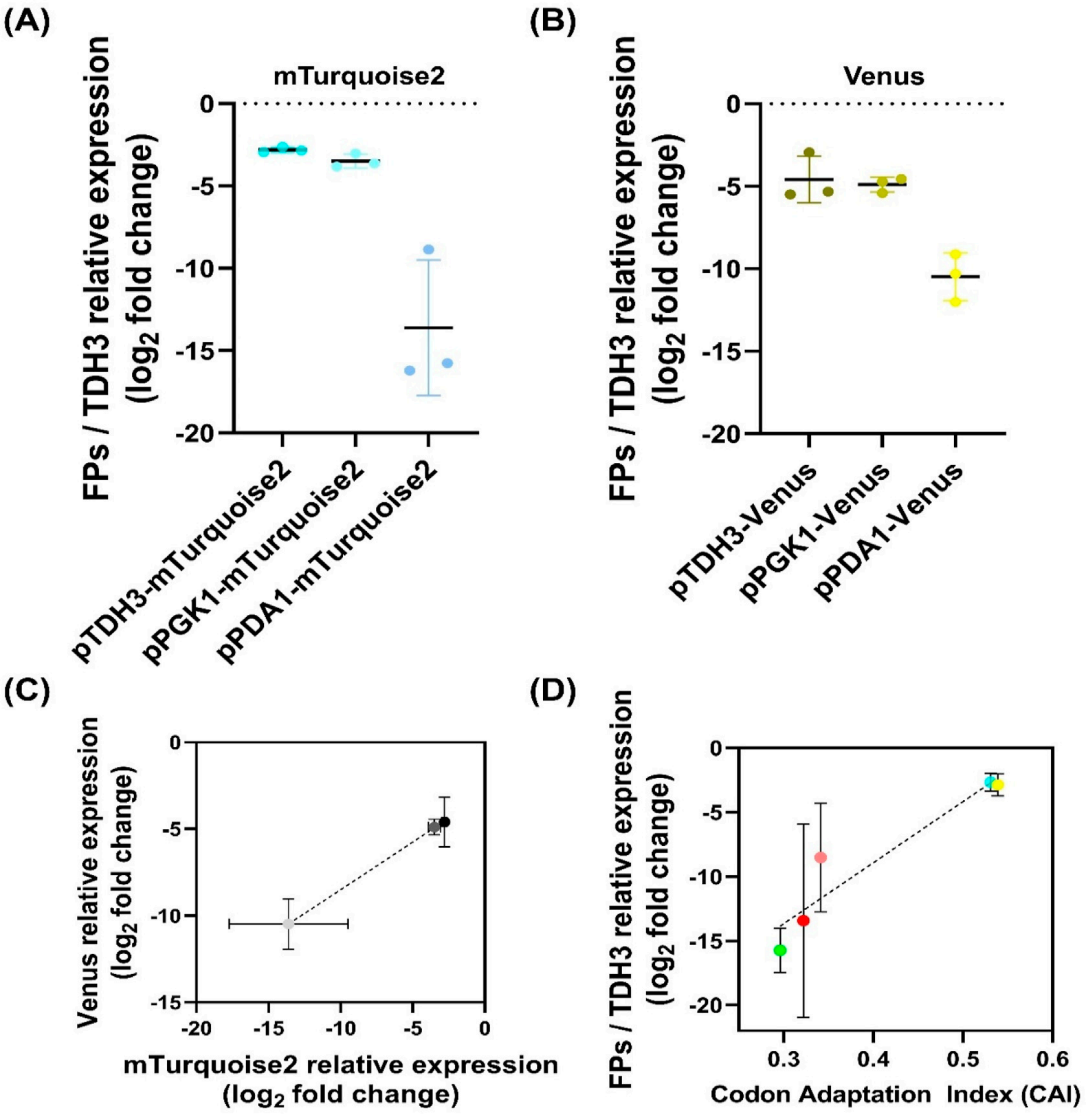
Promoter-dependent transcriptional expression and correlation analysis in *Wickerhamomyces ciferrii* **(A, B)** Promoter-dependent transcriptional expression of mTurquoise2 **(A)** and Venus **(B)** in *Wickerhamomyces ciferrii*. Expression levels were quantified by qPCR, normalized to TDH3, and presented as log_2_ fold change values. Data represent biological triplicates. **(C)** Correlation between promoter strength and transcriptional expression. The log_2_ fold change values of mTurquoise2 and Venus from **(A, B)** were plotted and analyzed by linear regression, confirming that promoter strength is independent of the downstream coding sequence **(D)** Correlation between transcriptional expression and codon adaptation index (CAI). The relative transcriptional expression levels of five fluorescent proteins were plotted against their CAI values and analyzed by linear regression to assess the influence of codon usage on gene expression.

The availability of stable endogenous promoters is essential for expanding the genetic toolkit in *W. ciferrii*, particularly for optimizing heterologous protein expression. To characterize promoter strength, we evaluated three native promoters—*PDA1* promoter (pPDA1), *PGK1* promoter (pPGK1), and *TDH3* promoter (pTDH3)— in a plasmid-based expression system. While pPDA1 and pPGK1 have been previously utilized, pTDH3 was selected for its strong constitutive activity in yeast (Lee et al., 2015; Mumberg et al., 1995; Redden and Alper, 2015; Xiong et al., 2018). We hypothesized that these promoters would exhibit differential expression levels (pTDH3 > pPGK1 > pPDA1) and tested their ability to drive fluorescent protein (FP) expression. Additionally, to assess promoter strength independent of the coding sequence effects, two highly transcribed FPs, mTurquoise2 and Venus, were expressed under each promoter using 2μ origin-based vectors (Figures 4A,B).

Expression analysis confirmed that pTDH3 drove the highest transcriptional levels, followed by pPGK1, while pPDA1 exhibited significantly lower transcriptional expression with higher colony-to-colony variation (Figures 4A,B). For mTurquoise2, pTDH3 expression was 1.6-fold higher than pPGK1 and 1,791-fold higher than pPDA1, while pPGK1 expression was 1,107-fold higher than pPDA1. Similar trends were observed for Venus, where pTDH3 was 1.2-fold higher than pPGK1 and 59.4-fold higher than pPDA1, while pPGK1 was 48.1-fold higher than pPDA1 (Figures 4A,B). To confirm that expression differences were due to promoter strength rather than coding sequence variation, a correlation analysis between mTurquoise2 and Venus transcription levels was performed (Figure 4C). Linear regression analysis revealed a strong correlation (R^2^ = 0.8841, *p* = 0.0002), indicating that promoter strength was the primary determinant of transcript abundance.

### Correlation between CAI values and transcriptional expression levels

To assess the impact of codon usage on transcript stability in *W. ciferrii*, we calculated codon adaptation index (CAI) values for the FPs and antibiotic resistance genes used in this study. The CAI values for the FPs were as follows: sfGFP (0.296), mTurquoise2 (0.531), Venus (0.539), mRuby2 (0.341), mKO (0.322), and mCherry (0.322). For antibiotic resistance genes, nourseothricin had a CAI of 0.463, zeocin 0.546, geneticin 0.293, and hygromycin B 0.140.

A positive correlation was observed between CAI values and transcriptional expression levels of FPs (Figure 4D). Consistent with qPCR results, mTurquoise2 and Venus exhibited the highest expression, while mRuby2 and mCherry showed moderate levels, and sfGFP displayed the lowest. Due to undetectable transcripts, mKO data were excluded.

Linear regression analysis confirmed a strong correlation between CAI values and FP/TDH3 expression (*R* ^*2*^ = 0.6551, *p* = 0.0003), suggesting that codon optimization significantly influences transcript abundance. Notably, *W. ciferrii* exhibited resistance to nourseothricin, zeocin, and geneticin but failed to grow under hygromycin B selection. The low CAI value of the hygromycin B resistance gene (0.140) suggests that poor codon adaptation may contribute to its inefficacy in *W. ciferrii*.

### Overexpression of *ACC*1 using the developed genetic toolbox in *Wickerhamomyces ciferrii* for enhanced TAPS production

To apply the developed genetic toolkit for improving tetraacetyl phytosphingosine (TAPS) production, we targeted *ACC1*, a key gene in fatty acid biosynthesis that may influence TAPS biosynthesis (Choi and Da Silva, 2014). The endogenous *ACC1* gene in *W. ciferrii* was identified via BLASTp using *R. norvegicus ACC1* as a query, revealing 46.5% overall amino acid identity. Conserved domain analysis confirmed the presence of essential catalytic regions, including the biotin carboxylation, biotin-binding, and carboxyltransferase domains, aligning with known *ACC*1 orthologs (Blum et al., 2025) (Supplementary Data S1). Since ACC1 activity is regulated by phosphorylation, we examined conserved inhibitory sites in *Rattus norvegicus ACC1* (Davies et al., 1990; Ha et al., 1994) and identified two functionally conserved serine residues (S26 and S1161) in *W. ciferrii*. To generate a constitutively active variant, we introduced S26 A and S1161 A mutations via site-directed mutagenesis, producing *ACC1* ^*S26A-S1161A*^.

**FIGURE 5:**
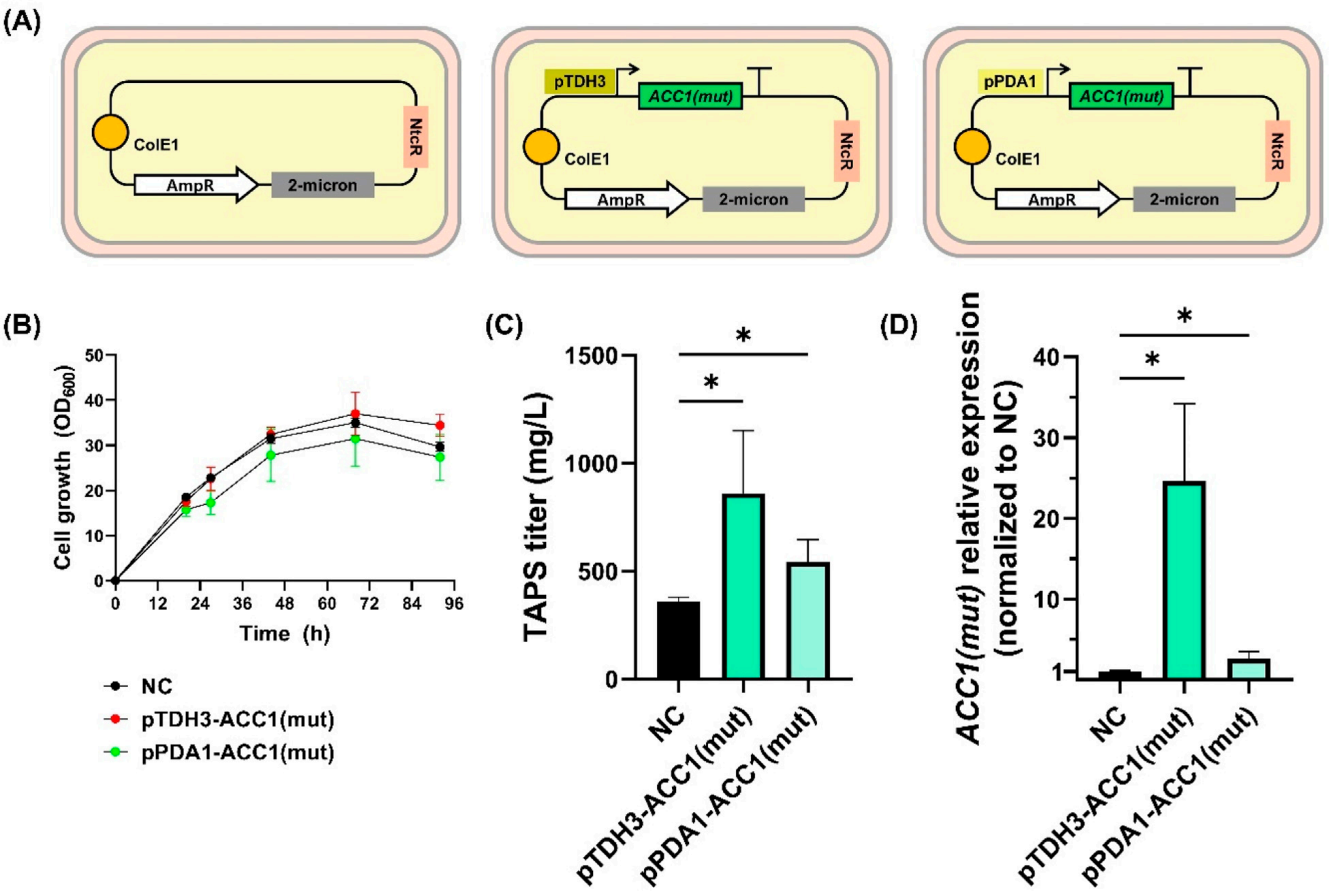
Growth and TAPS production of *Wickerhamomyces ciferrii* expressing mutant *ACC1* ^*S26A-S1161A*^ **(A)** Schematic representation of plasmid constructs used in this study. *Wickerhamomyces ciferrii* strains were transformed with either an empty vector (*W.c_2μ-ev*), a plasmid expressing *ACC1* ^*S26A-S1161A*^ under the strong *TDH3* promoter (*W.c_2μ-ACC1m-str*), or a plasmid expressing *ACC1* ^*S26A-S1161A*^ under the weak *PDA1* promoter (*W.c_2μ-ACC1m-weak*). **(B)** Growth curves of the three strains cultured in YMglSCN medium at 25°C with shaking at 250 rpm. Optical density at 600 nm (OD_600_) was measured at regular intervals. **(C)** TAPS titers after 96 h of shake-flask cultivation **(D)** Relative transcriptional expression of mutant *ACC1* ^*S26A-S1161A*^ in each strain, normalized to *TDH3* expression and subsequently to the empty vector control (*W.c_2μ-ev*). Statistical significance was determined using an unpaired *t*-test with Welch’s correction (*p* < 0.05). Error bars represent standard deviations from biological replicates.

This mutant was cloned into a 2μ based plasmid carrying a nourseothricin resistance marker under either the strong pTDH3 promoter or the weak pPDA1 promoter, generating p2μ_pTDH3-ACC1(mut)_NTC and p2μ_pPDA1-ACC1(mut)_NTC. The resulting plasmids were transformed into *W. ciferrii*, generating three recombinant strains: *W. c_2μ-ACC1m-str* (with pTDH3), *W. c_2μ-ACC1m-weak* (with pPDA1), and *W. c_2μ-ev* (empty vector control) (Figure 5A). These strains were cultured in YMglSCN medium at 25°C and 250 rpm for 96 h to assess growth and TAPS production.

Growth analysis revealed that *W. c_2μ-ev* strain reached an OD_600_ of 29.6, while *W. c_2μ-ACC1m-str* and *W. c_2μ-ACC1m-weak* reached 34.4 and 27.4, respectively (Figure 5B). Notably, overexpression of *ACC1* ^*S26A-S1161A*^ significantly enhanced TAPS production compared to the control (Figure 5C). Average TAPS titers were 359.0 ± 20.3 mg/L in *W. c_2μ-ev*, 860.8 ± 291.5 mg/L in *W. c_2μ-ACC1m-str* (2.4-fold increase, *p* < 0.05), and 542.4 ± 104.5 mg/L in *W. c_2μ-ACC1m-weak* (1.5-fold increase, *p* < 0.05). qPCR analysis confirmed that *ACC1* ^*S26A-S1161A*^ expression correlated with TAPS titers, with pTDH3-driven expression yielding a 24.6-fold increase, whereas pPDA1 resulted in a 2.6-fold increase, both significantly higher than the control (*p* < 0.05) (Figure 5D).

Overall, these results demonstrate that *ACC1* ^*S26A-S1161A*^ overexpression enhances TAPS production in *W. ciferrii*, with the strong pTDH3-driven expression yielding the highest titers. These findings validate the developed genetic tools for strain engineering and establish *ACC1* engineering as a viable strategy for spingoid base biosynthetic pathway optimization.

## Discussion

This study established a stable episomal plasmid system for *W. ciferrii*, making a significant advancement in enabling efficient genetic modifications in this non-model yeast. We demonstrated that *S. cerevisiae*-derived replication origins, 2μ and CEN6/ARS4, support plasmid replication and maintenance in *W. ciferrii*, with the 2μ origin yielding a higher copy number and lower colony-to-colony variation. These findings provide a versatile platform for genetic engineering applications, facilitating rapid strain development for metabolic engineering and synthetic biology.

Despite successful episomal plasmid maintenance, the measured 2μ plasmid copy number in *W. ciferrii* was significantly lower than in *S. cerevisiae,* averaging only 1–2 copies per cell. In *S. cerevisiae*, native 2μ plasmids are typically maintained at 40–80 copies per haploid cell, though copy numbers drop to 8–14 copies when carrying selective markers (Clark-Walker and Miklos, 1974; Futcher, 1986; Futcher and Cox, 1984; Gerbaud and Guérineau, 1980; Gnügge and Rudolf, 2017; Karim et al., 2013). This discrepancy is likely due to the absence of the Flp/FRT recombination system in *W. ciferrii*, which amplifies 2μ plasmid copy number in *S. cerevisiae* through site-specific recombination (Chan et al., 2013; Gronostajski and Sadowski, 1985). Without this amplification mechanism, *W. ciferrii* may rely solely on passive plasmid replication, leading to a lower plasmid copy number. Future studies could explore *W. ciferrii*-specific centromere sequences to develop native CEN-based replication systems for stable plasmid inheritance without selection pressure (Cao et al., 2017).

Our evaluation of selectable markers identified nourseothricin, zeocin, and geneticin as reliable selection agents, whereas hygromycin B failed to confer resistance. The failure is likely due to hygromycin B resistance gene’s low codon adaptation index (CAI) (0.140), suggesting inefficient expression in *W. ciferrii*. These findings underscore the importance of codon optimization when designing selection markers for non-model yeasts. Previous studies have demonstrated that codon optimization can drastically enhance gene expression in yeasts. For example, Kaishima et al. showed that codon-optimized GFP variants exhibited over 100-fold higher fluorescence in *S. cerevisiae* compared to non-optimized versions (Kaishima et al., 2016). Similarly, Gordon et al. reported that codon adaptation of selectable markers was essential for functional expression in non-model yeasts such as *Metschnikowia borealis*, where non-optimized versions failed to produce transformants (Gordon et al., 2019). These findings suggest that codon-optimized versions of fluorescent proteins and resistance markers may also yield improved performance in *W. ciferrii*.

Additionally, we observed that higher antibiotic concentrations were required for selection on solid media compared to liquid cultures, likely due to differences in antibiotic diffusion and agar binding, reducing bioavailability. Similar challenges in antibiotic efficacy have been observed in structured environments, where factors such as metabolic dormancy and limited diffusion reduce the effectiveness of antibiotics (Stokes et al., 2019). Furthermore, cell debris has been shown to bind and sequester antibiotics, further diminishing their activity in solid-phase conditions (Yuan et al., 2022). These findings underscore the importance of media-specific antibiotic optimization for robust selection in W. ciferrii genetic engineering.

Expanding the fluorescent protein (FP) toolkit for *W. ciferrii*, we validated the expression of mTurquoise2, Venus, mRuby2, and mCherry, in addition to the previously reported GFP. These tools enable multi-gene expression tracking, subcellular localization studies, and high-throughput screening (HTS). Fluorescent reporters facilitate pathway engineering by allowing real-time monitoring of enzyme expression and metabolic flux adjustments. Additionally, fluorescence-activated cell sorting (FACS) can enhance strain selection and single-cell heterogeneity analysis, improving metabolic pathway refinement. Interestingly, significant transcriptional variability was observed among different FPs expressed under the same promoter. While promoter strength is a key determinant of transcript abundance, codon adaptation showed a strong correlation with expression levels (*R* ^*2*^ = 0.6551, *p* = 0.0003), suggesting that codon bias affects both mRNA stability and translational efficiency (Brule and Grayhack, 2017; Presnyak et al., 2015). This effect may be exacerbated by the low genomic G + C content (30.4%) of *W. ciferrii*, which influences mRNA folding, ribosome binding, and protein synthesis efficiency (Schneider et al., 2012). The failure of the hygromycin B resistance gene further supports this hypothesis, emphasizing the necessity of codon optimization for heterologous gene expression in *W. ciferrii*.

Overexpression of *ACC1* ^S26A−S1161A^ significantly increased TAPS production, confirming that acetyl-CoA availability is a key limiting factor in sphingoid base biosynthesis. However, despite higher *ACC1* transcription levels compared to control strains, the increase in TAPS production was relatively modest between strong (pTDH3) and weak (pPDA1) promoter-driven strains, suggesting the presence of additional metabolic bottlenecks. Previous studies have shown that overexpression of *LCB1, LCB2*, and *SYR2* enhances sphingoid base production (Schorsch et al., 2012), indicating that a combinatorial approach integrating *ACC1* activation with pathway gene co-expression may be more effective. Future research should focus on optimizing metabolic flux balancing strategies to further enhance TAPS yields.

Despite successful episomal expression, colony-to-colony variation remained a challenge, with substantial heterogeneity in FP expression across individual cells. This variability likely results from differences in plasmid copy number or transcriptional stability, necessitating rigorous clone selection before downstream experiments. Genome integration may provide a more stable alternative, ensuring consistent gene expression (Zhao et al., 2020). The higher transformation efficiency further supports genome integration as a viable alternative for stable gene expression. Advances in CRISPR-based genome editing could enable precise targeted gene integration, reducing variability and improving genetic stability in engineered *W. ciferrii* strains (Cai et al., 2019; Raschmanová et al., 2018).

Beyond its use in TAPS biosynthesis, *W. ciferrii* and its genetic components also hold promise for broader synthetic biology applications. Notably, the *W. ciferrii* α-mating factor secretion signal has demonstrated superior efficiency compared to the *S. cerevisiae* α-factor when used in *Komagataella phaffii*, enhancing heterologous protein secretion (Zou et al., 2022). This enhanced secretion observed in *K. phaffii* suggests that *W. ciferrii* may possess an inherently strong secretory system. Accordingly, *W. ciferrii* itself could be engineered as a chassis for industrial protein production, particularly for lipid-associated or membrane proteins that may benefit from its native metabolic context. Moreover, the high-level production of TAPS implies a robust acetyl-CoA and fatty acid precursor pool. This metabolic capacity suggests potential for the biosynthesis of a broader range of lipid-derived compounds, including ceramides, long-chain fatty alcohols, and other hydrophobic molecules. The combination of strong lipid-handling capacity and the versatile genetic tools developed in this study positions *W. ciferrii* as a valuable yet underutilized host for industrial lipid biotechnology.

In conclusion, this study established a versatile genetic toolkit for *W. ciferrii*, including a robust episomal plasmid system, optimized selection markers, and an expanded set of functional fluorescent proteins. Our findings highlight the importance of codon adaptation in heterologous gene expression and validate *ACC1* ^*S26A-S1161A*^ overexpression as a strategy to enhance TAPS biosynthesis. Future efforts should focus on refining genome editing strategies using CRISPR-based targeted gene manipulation, optimizing metabolic pathways through combinatorial gene expression approaches, and enhancing episomal plasmid retention by leveraging native replication elements in *W. ciferrii*. Together, these advancements not only position *W. ciferrii* as a genetically accessible non-conventional yeast but also reinforce its industrial relevance as a platform for scalable bioproduction and cross-system synthetic biology applications.

## Data Availability

The datasets presented in this study can be found in online repositories. The names of the repository/repositories and accession number(s) can be found in the article/Supplementary Material.
