# Supplementary material for: A versatile genetic toolkit for engineering Wickerhamomyces ciferrii for tetraacetyl phytosphingosine production
Source: Front Bioeng Biotechnol. 2025 Apr 28;13:1586218. doi: 10.3389/fbioe.2025.1586218 (PMC12066694; doi:10.3389/fbioe.2025.1586218)
Supplement: Supplementary file 1 [file DataSheet1.docx]

**Supplementary Data 1**


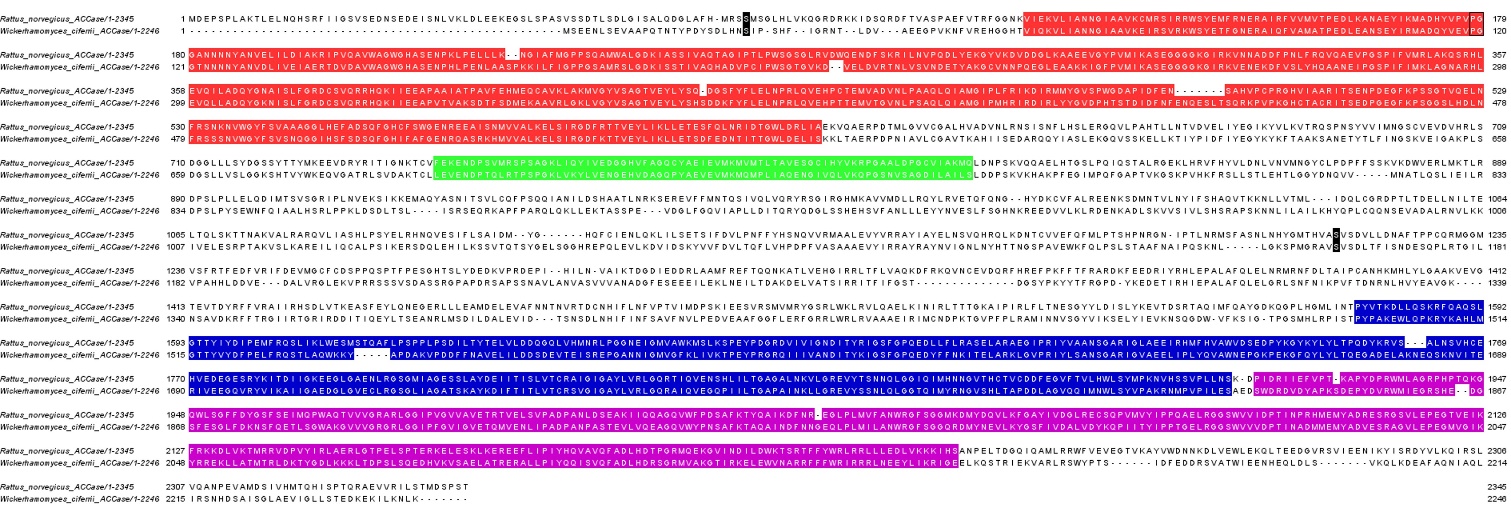


**Supplementary Data 1. Sequence alignment of *Rattus norvegicus* and *Wickerhamomyces ciferrii* ACC1 amino acid sequences.** The amino acid sequence of *Wickerhamomyces ciferrii* ACC1 was aligned with *Rattus norvegicus* ACC1, revealing an overall sequence identity of 46.5%. Conserved functional domains are highlighted as follows: biotin carboxylation domain (red, 66.35% identity), biotin-binding domain (green, 52.00% identity), N-terminal carboxyltransferase domain (blue, 55.08% identity), and C-terminal carboxyltransferase domain (purple, 55.49% identity). The serine residues targeted for mutation (*S26A* and *S1161A*), which prevent enzymatic inhibition via phosphorylation, are highlighted in black.

**Supplementary Data 2**


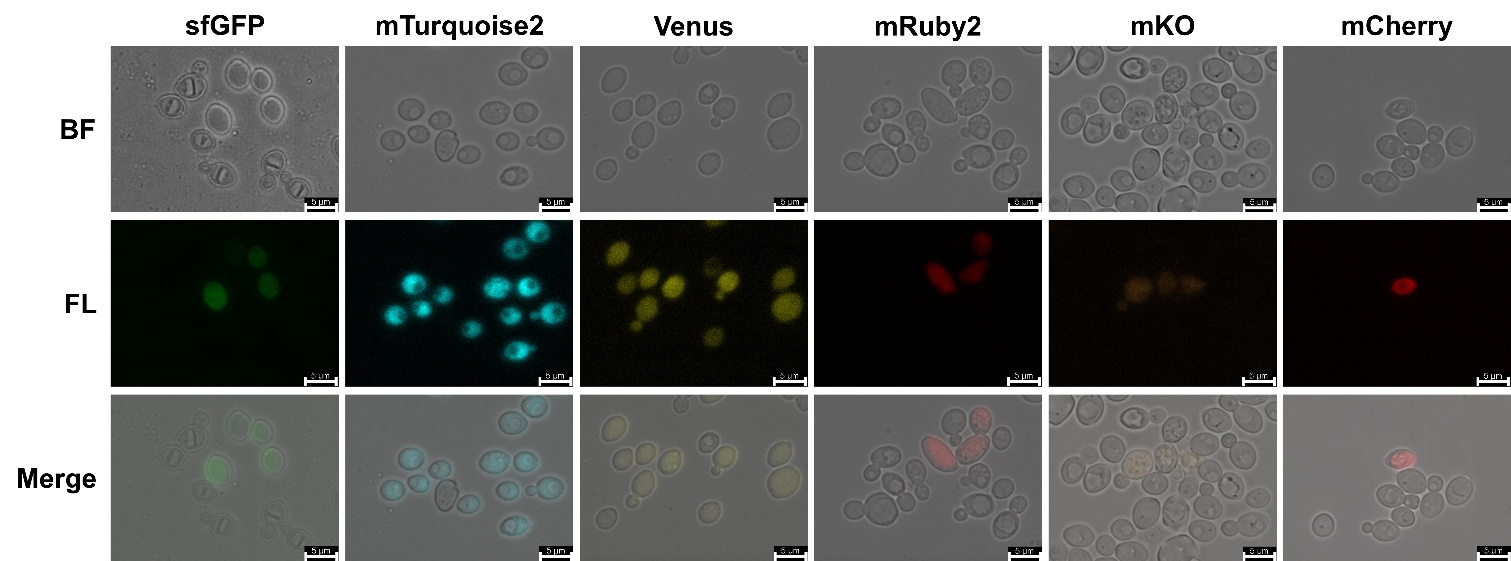


**Supplementary Data 2. Fluorescence microscopy images of *W. ciferrii* transformants expressing different fluorescent proteins from CEN6/ARS4-based vectors.** Cells harboring plasmids encoding sfGFP, mTurquoise2, Venus, mRuby2, mKO, or mCherry were imaged under bright-field (BF) and fluorescence (FL) microscopy, with merged images provided for comparison. Fluorescent proteins were expressed under the *PGK1* promoter, and cells were imaged at mid-log phase following cultivation in YPDN medium (YPD supplemented with 50 μg/mL nourseothricin). Representative single-cell fluorescence images are shown to visualize protein-level expression from CEN6/ARS4-based vectors. Scale bars, 5 μm.
